# Oral anticoagulation in patients with gastrointestinal bleeding and new‐onset atrial fibrillation: A population‐based registry‐linkage study

**DOI:** 10.1111/joim.70018

**Published:** 2025-09-23

**Authors:** Santeri Jolkkonen, Jukka Putaala, Konsta Teppo, Pirjo Mustonen, Jussi Jaakkola, Aapo Aro, Olli Halminen, Ossi Lehtonen, Jari Haukka, Miika Linna, Juha Hartikainen, K. E. Juhani Airaksinen, Mika Lehto

**Affiliations:** ^1^ Faculty of Medicine University of Helsinki Helsinki Finland; ^2^ Department of Gastroenterology and Alimentary Tract Surgery Tampere University Hospital Tampere Finland; ^3^ Department of Neurology HUS Helsinki University Hospital and University of Helsinki Helsinki Finland; ^4^ Heart Center Turku University Hospital and University of Turku Turku Finland; ^5^ Heart Unit, Satasairaala Pori Finland; ^6^ Heart and Lung Center HUS Helsinki University Hospital and University of Helsinki Helsinki Finland; ^7^ Department of Industrial Engineering and Management Aalto University Espoo Finland; ^8^ Department of Health and Social Management University of Eastern Finland Kuopio Finland; ^9^ Department of Public Health Clinicum University of Helsinki Helsinki Finland; ^10^ Heart Center Kuopio University Hospital and University of Eastern Finland Kuopio Finland; ^11^ Department of Internal Medicine Jorvi Hospital, HUS Helsinki University Hospital Espoo Finland

**Keywords:** anticoagulants, atrial fibrillation, gastrointestinal hemorrhage, retrospective studies

## Abstract

**Background:**

Limited data exist on the prevalence of gastrointestinal bleeding (GIB) in patients with new‐onset atrial fibrillation (AF) and the impact of GIB on the initiation of oral anticoagulation (OAC) therapy.

**Methods:**

A population‐based registry‐linkage study included all patients diagnosed with new‐onset AF in Finland during 2010–2018 who had available laboratory data and a definite indication for OAC therapy. The primary outcome was OAC initiation within 90 days following AF diagnosis. Factors associated with OAC initiation were assessed using modified Poisson regression.

**Results:**

Among 117 997 patients with new‐onset AF, 6628 (5.6%) had GIB, of which 5336 occurred more than 30 days prior to AF diagnosis, and 1292 were temporally (±30 days) associated with new‐onset AF (GIBTAF). Patients with GIB compared to those without GIB were older (mean age 78.3 vs. 75.3 years), more frequently men (48.5% vs. 41.9%), and had more comorbidities. The occurrence of GIB was associated with a lower probability of initiating OAC (RR 0.84, 95% CI 0.81–0.86). Among patients with GIB, an obscure origin of GIB (RR 0.93, 95% CI 0.88–0.99) or GIBTAF reduced the likelihood of OAC initiation (RR 0.72, 95% CI 0.66–0.79). The initiation of OAC did not depend on the known GIB bleeding site (lower vs. upper). Overall, the initiation of OAC therapy increased from 2010 to 2018 but remained consistently lower in patients with GIB.

**Conclusion:**

Prior and concurrent GIB is common among patients with new‐onset AF, and despite the overall increasing use of OACs, they remain less utilized in patients with GIB.

## Graphical abstract

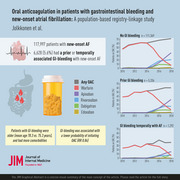

## Introduction

Atrial fibrillation (AF) is a prevalent heart rhythm disorder necessitating in most patients a lifelong oral anticoagulation (OAC) treatment to mitigate the risk of stroke [1]. The principal challenges associated with OAC therapy lie in the risk of bleeding complications [2], of which gastrointestinal bleeding (GIB) is the most frequent complication, manifesting in 5%–15% of patients with long‐term OAC use [3]. Particularly, in the aging population, the prevalence of AF, the requirement for OAC treatment, and consequently, the incidence of GIB are anticipated to rise [1].

The risk factors for ischemic stroke and GIB in AF patients are well established [4, 5, 6]. Given the diverse etiology and severity of GIB [7], evaluating the benefit of OAC therapy in patients with prior GIB poses a challenge in balancing the risk of stroke against the risk of bleeding, particularly as those at high stroke risk are often also predisposed to risk of bleeding [8]. Real‐world data suggest varying risks of GIB associated with different anticoagulants [9], and it is presumable that the history or the presence of GIB affects the choice of anticoagulant when initiating OAC therapy. Despite the high prevalence of GIB [10], limited data exist on the impact of GIB on the initiation of OAC therapy in patients with new‐onset AF [11].

In this nationwide, population‐based cohort study, we evaluated the initiation of OAC treatment and the choice of anticoagulant in patients with new‐onset AF with and without a history of GIB. Furthermore, considering the diversity of bleeding sites and varying risks of rebleeding, we assessed whether the site of bleeding and its temporal proximity to AF onset influenced OAC initiation.

## Methods

### Study population

The Finnish Anticoagulation in Atrial Fibrillation (FinACAF) Study (ClinicalTrials Identifier: NCT04645537; ENCePP Identifier: EUPAS29845) is a nationwide retrospective cohort study including all diagnosed AF patients in Finland during 2004–2018. Patients were identified from all national health care registers: HILMO (hospitalizations and outpatient specialist visits), AvoHILMO (primary healthcare), and KELA (National Reimbursement Register maintained by the Social Insurance Institute) [12]. Laboratory data were obtained from the six largest national central laboratories.

Inclusion criterion for the FinACAF database was a diagnosis code I48 from the International Classification of Diseases, Tenth Revision (ICD‐10), recorded between 2004 and 2018, representing AF and atrial flutter (collectively referred as AF). Cohort entry was marked by the date of the first recorded AF diagnosis, with follow‐up extending to death or December 31, 2018, whichever occurred first. A total of 411 387 AF patients were identified.

This sub‐study focused on patients with new‐onset AF who had both available laboratory data and a definite indication for OAC therapy. Indication was defined as a CHA_2_DS_2_–VASc score of ≥2 for men and ≥3 for women, considering the components of the score: congestive heart failure, hypertension, age ≥75 years, diabetes, history of stroke or transient ischemic attack, vascular disease, age 65–74 years, and female sex. To exclude patients with possible previous AF diagnoses, patients with fulfilled warfarin prescriptions during 2004–06 were excluded due to insufficient look‐back time. Similarly, patients with a fulfilled OAC prescription in the year preceding the index date were excluded. Because laboratory data were available only after January 2010, patients with cohort entry before 2010 were not included. Focusing on patients with a definite indication for OAC, patients at low or intermediate risk of stroke were also excluded. To capture OAC initiation within 90 days after AF diagnosis, those with inadequate follow‐up (cohort entry after August 31, 2018) were excluded. Consequently, 117 997 patients were included in the current analysis (Fig. S1).

**Fig. 1:**
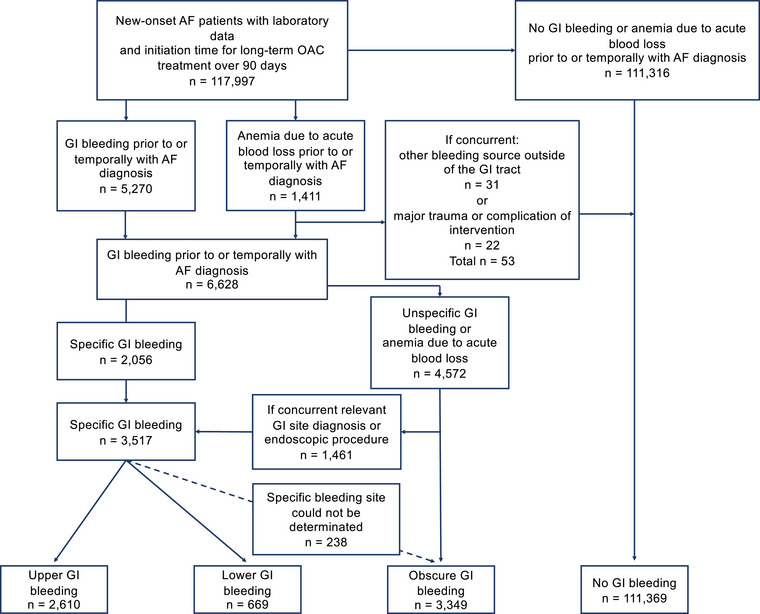
Flowchart illustrating the groups of gastrointestinal bleeding based on the bleeding site. AF, atrial fibrillation; GI, gastrointestinal; OAC, oral anticoagulation.

The definitions of the baseline characteristics are displayed in Table S1. Laboratory results prior to and closest to the cohort entry, including hemoglobin, platelet count, and estimated glomerular filtration rate (eGFR) calculated from serum creatinine level using the Chronic Kidney Disease Epidemiology Collaboration equation, were included for analysis. Anemia was defined according to the reference values of the Finnish population for both genders.

### Definition of gastrointestinal bleeding

Episodes of GIB were identified from the hospital register (Finland Care Register for Health Care. In finnish “Terveydenhuollon hoitoilmoitusrekisteri (HILMO)”) using the ICD‐10 codes indicating GIB. Furthermore, episodes of anemia due to acute blood loss, as defined by ICD‐10 codes, were attributed to GIB after excluding bleeding from other sites, major trauma, or procedural complications during the same hospital visit. For patients with multiple GIB episodes, the closest episode to AF diagnosis was considered for analysis.

Based on the temporal association with the AF cohort entry, GIBs were categorized as (1) either prior GIB if GIB occurred more than 30 days before AF onset or (2) GIB temporally associated with new‐onset AF (GIBTAF) if GIB occurred within ±30 days from AF diagnosis. Both categories were referred to as GIB.

Moreover, patients with GIB were categorized into three groups based on the bleeding site: upper GIB (UGIB)—proximal to the ligament of Treitz; lower GIB (LGIB)—distal to the ligament of Treitz; or obscure GIB (OGIB). GIB site was determined using the ICD‐10 codes and the Nordic Medico‐Statistical Committee Classification of Surgical Procedures (NCSP) codes. Patients without a specific diagnosis code for GIB, or concurrent secondary diagnosis code or endoscopic procedure implying a GIB site, were categorized as OGIB. If multiple diagnostic or procedural codes for GIB were available, the most clinically significant code was prioritized. If a clear classification could not be assigned, the patient was categorized as OGIB. The categorization process is visualized in Fig. 1, and the ICD‐10 and the NCSP codes used for categorization are listed in Table S2.

### Primary outcome

The primary outcome was the initiation of OAC therapy within 90 days following cohort entry, marked by the date of the first fulfilled prescription for OAC (warfarin, apixaban, dabigatran, edoxaban, or rivaroxaban). The 90‐day mark was chosen to capture the true initiation of OAC, which could otherwise be underestimated immediately after an AF diagnosis due to short‐term low‐molecular‐weight heparin treatment or delayed initiation of OAC following an acute illness.

### Study ethics

Approval for the study protocol was obtained from the Ethics Committee of the Medical Faculty at Helsinki University, Helsinki, Finland (nr. 15/2017), with research permission granted by the Helsinki University Hospital (HUS/46/2018). Necessary permissions were also acquired from Finnish register holders (KELA 138/522/2018; THL 2101/5.05.00/2018; Population Register Centre VRK/1291/2019‐3; Statistics Finland TK‐53‐1713‐18/u1281; and Tax Register VH/874/07.01.03/2019), as well as from the hospital districts for the laboratory results. All patient data underwent pseudonymization to ensure full compliance with the European General Data Protection Regulation. Considering the study's retrospective registry nature, the requirement for informed consent was waived. The study conforms to the Declaration of Helsinki as revised in 2013.

### Statistical analysis

Continuous variables with a normal distribution were reported as mean (standard deviation), and those with a non‐normal distribution were reported as median (interquartile range). To assess factors associated with OAC initiation after new‐onset AF in the entire study cohort and among patients with GIB, a modified Poisson regression model was employed. This approach was selected due to the high incidence of the primary outcome (OAC initiation) in the study population [13, 14]. The model was adjusted for demographics, annual income, comorbidities, medication use, cancer status, laboratory results, and attributes of GIB. Results were reported as relative risk with 95% CI. Assumptions for the model were assessed visually, and multicollinearity was tested. We used age (<65, 65–74, and ≥75 years), annual income (quartiles), hemoglobin level (anemia and normal), platelet count (≤100 and >100 mcL), eGFR (<15, 15–29, 30–59, and ≥60 mL/min/1.73 m^2^), GIB subgroups (UGIB, OGIB, and LGIB), and time between GIB and AF onset (±30 and >30 days) as categorical variables. Patients with missing data were excluded from the regression analysis. All variables were subsequently forced into the model. We performed a similar sensitivity analysis in the entire study cohort by excluding patients with GIB and among patients with GIB by eliminating the attributes of GIB. Statistical analyses were performed with R (version 2023.03.0 + 386, https://www.R‐project.org).

## Results

Among the 117 997 patients with new‐onset AF, we identified 6628 patients (5.6%) with GIB. GIB patients were categorized into two groups based on the temporal association with the initial AF episode: prior GIB (5336 [80.5%]) and GIBTAF (1292 [19.5%]). Additionally, based on the location of bleeding, GIBs were divided into three categories: UGIB (2610 [39.4%]), LGIB (669 [10.1%]), and OGIB (3349 [50.5%]) (Fig. 1).

Patients with GIB were older, more frequently male, and had substantially more comorbidities (Table 1). The prevalence of previous cancer was higher among patients with GIB (25.6% vs. 19.5%), with luminal gastrointestinal tract cancers being particularly common (6.5% vs. 1.9%). Furthermore, patients with GIB had lower mean hemoglobin levels and eGFR when compared to patients without GIB.

**Table 1:** Baseline characteristics of the study cohort according to gastrointestinal bleeding status and site.

			GIB subgroups		
	No GIB	GIB	Upper	Lower	Obscure
Variable	(*n* = 111 369)	(*n* = 6628)	(*n* = 2610)	(*n* = 669)	(*n* = 3349)
**Demographics and socioeconomic status**					
Age, years	75.3 (11.0)	78.3 (9.9)	77.1 (9.7)	78.5 (9.3)	79.1 (10.2)
Male sex	46 704 (41.9)	3217 (48.5)	1410 (54.0)	301 (45.0)	1506 (45.0)
Annual taxable income, 1000 €	13.0 (4.0–29.0)	10.0 (3.0–23.0)	11.0 (3.0–24.8)	11.0 (3.0–25.0)	9.0 (2.0–21.0)
**Risk scores**					
CHA_2_DS_2_–VASc score	3.9 (1.6)	4.5 (1.6)	4.3 (1.6)	4.4 (1.6)	4.6 (1.6)
Modified HAS‐BLED score	2.3 (0.9)	3.4 (1.0)	3.5 (1.0)	3.3 (0.9)	3.4 (1.0)
**Comorbidities**					
Prior bleeding	8545 (7.7)	6219 (93.8)	2485 (95.2)	652 (97.5)	3082 (92.0)
Hypertension	91 636 (82.3)	5741 (86.6)	2275 (87.2)	577 (86.2)	2889 (86.3)
Hyperlipidemia	62 534 (56.2)	3957 (59.7)	1567 (60.0)	405 (60.5)	1985 (59.3)
Diabetes	29 220 (26.2)	2118 (32.0)	851 (32.6)	203 (30.3)	1064 (31.8)
Coronary heart disease	28 890 (25.9)	2482 (37.4)	951 (36.4)	240 (35.9)	1291 (38.5)
Congestive heart failure	21 132 (19.0)	1994 (30.1)	751 (28.8)	168 (25.1)	1075 (32.1)
Ischemic stroke or TIA	20 470 (18.4)	1532 (23.1)	562 (21.5)	139 (20.8)	831 (24.8)
Venous thromboembolism	8540 (7.7)	740 (11.2)	269 (10.3)	84 (12.6)	387 (11.6)
Dementia	6457 (5.8)	576 (8.7)	211 (8.1)	47 (7.0)	318 (9.5)
Abnormal kidney function	5241 (4.7)	740 (11.2)	293 (11.2)	62 (9.3)	385 (11.5)
Abnormal liver function	510 (0.5)	198 (3.0)	110 (4.2)	2 (0.3)	86 (2.6)
Psychiatric disease	16 254 (14.6)	1459 (22.0)	559 (21.4)	112 (16.7)	788 (23.5)
Alcohol abuse	3906 (3.5)	597 (9.0)	270 (10.3)	24 (3.6)	303 (9.0)
**Medication**					
Antiplatelet or NSAID	34 474 (31.0)	2011 (30.3)	796 (30.5)	204 (30.5)	1011 (30.2)
SSRI	6475 (5.8)	484 (7.3)	165 (6.3)	45 (6.7)	274 (8.2)
PPI	59 480 (53.4)	5487 (82.8)	2329 (89.2)	521 (77.9)	2637 (78.7)
**Cancer status**					
Current cancer	5258 (4.7)	476 (7.2)	178 (6.8)	64 (9.6)	234 (7.0)
Previous cancer	21 681 (19.5)	1700 (25.6)	576 (22.1)	231 (34.5)	893 (26.7)
Lower GI tract cancer	1809 (1.6)	341 (5.1)	63 (2.4)	97 (14.5)	181 (5.4)
Upper GI tract cancer	314 (0.3)	95 (1.4)	63 (2.4)	5 (0.7)	27 (0.8)
**Laboratory results**					
Hemoglobin (g/L)	134 (18)	124 (21)	125 (21)	126 (19)	123 (21)
Platelet count (mcL)	238 (83)	244 (103)	241 (98)	248 (105)	246 (107)
eGFR (mL/min/1.73 m^2^)	70 (21)	65 (24)	66 (24)	66 (23)	64 (24)

*Note*: Quantitative variables are presented as mean (standard deviation), except for annual income, which is non‐normally distributed and is presented as median (interquartile range). Categorical variables are presented as count (percentage). GIB: gastrointestinal bleeding; CHA_2_DS_2_–VASc, congestive heart failure, hypertension, age ≥75 years, diabetes, history of stroke or TIA, vascular disease, age 65–74 years, sex category (female); modified HAS‐BLED score, hypertension, abnormal renal or liver function, prior stroke, bleeding history, age >65 years, alcohol abuse, concomitant antiplatelet/NSAIDs (no labile INR, max score 8).

Abbreviations: eGFR, estimated glomerular filtration rate; NSAID, nonsteroidal anti‐inflammatory drug; PPI, proton‐pump inhibitor; SSRI, selective serotonin reuptake inhibitor; TIA, transient ischemic attack.

**Fig. 2:**
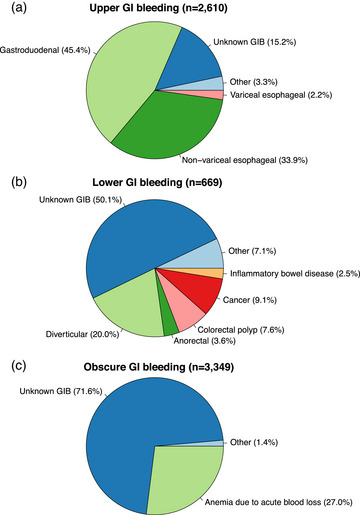
Distribution of presumed etiologies of gastrointestinal bleeding (GIB) in the three main GIB categories, (a) Upper GI bleeding; (b) Lower Gi Bleeding; (c) Obscure GI Bleeding. In the absence of an exact diagnosis code for GIB, the bleeding source was assessed based on the secondary diagnosis codes of the GIB episode, if available.

A comparative analysis of GIB subgroups highlighted that patients with LGIB less often had a history of alcohol use disorder or abnormal liver function and more often a prior cancer diagnosis compared to patients with UGIB or OGIB (Table 1). Episodes of GIB were temporally closest to the AF onset among patients with obscure GIB (the proportion of GIBTAF was 22.0% in OGIB, 18.4% in UGIB, and 11.2% in LGIB). Endoscopy was performed during GIB events at distinct rates: 69.5% in LGIB, 54.5% in UGIB, and 1.8% in OGIB. Fig. 2 shows the distribution of GIB sites across various GIB categories. Tables S3–S8 summarize the numbers of diagnoses and procedure codes in various GIB categories.

**Fig. 3:**
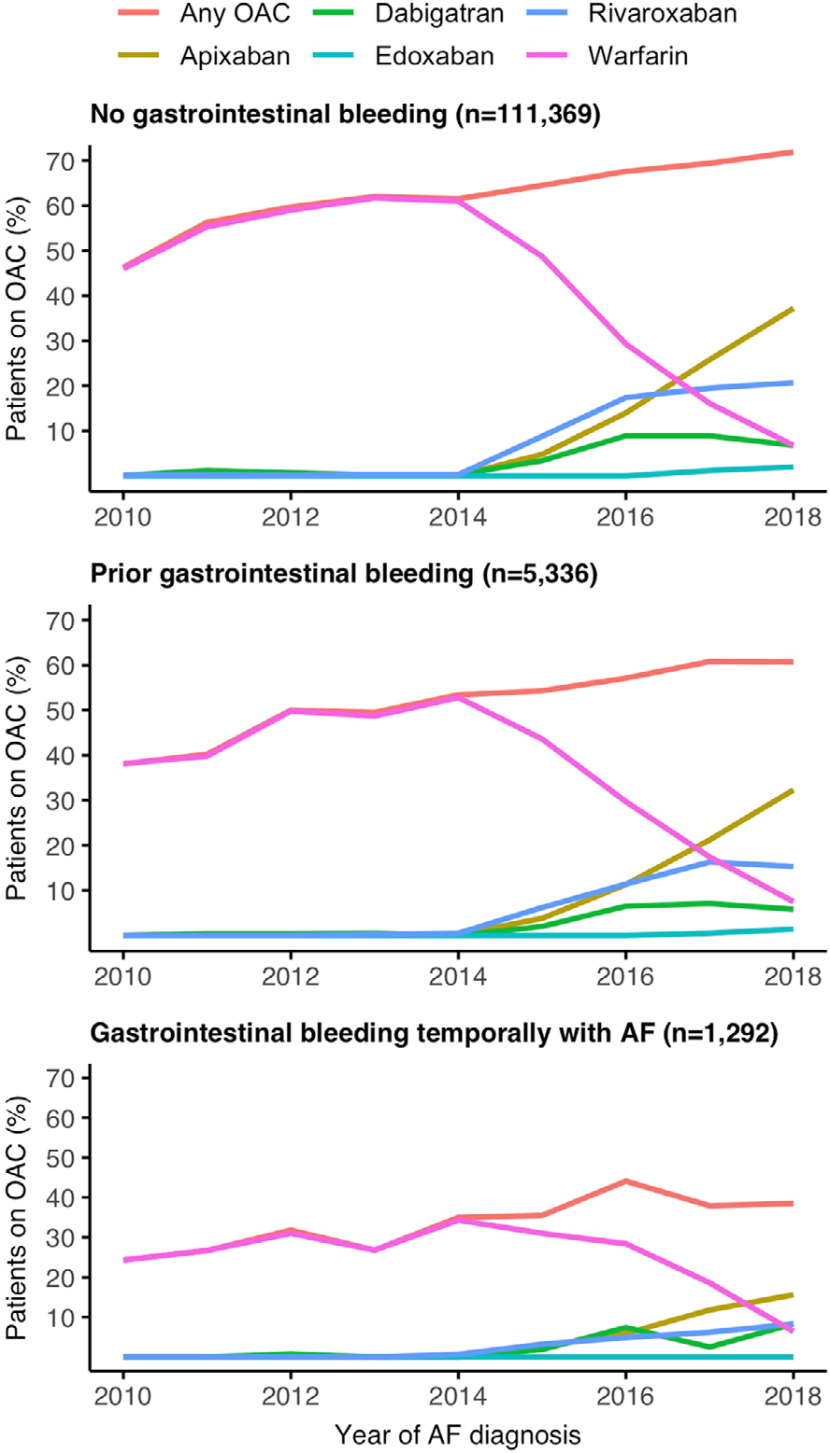
Temporal trends in the initiation of oral anticoagulation (OAC) therapy within 90 days following the diagnosis of atrial fibrillation (AF) from 2010 to 2018 delineated by the occurrence and timing of gastrointestinal bleeding events relative to the atrial fibrillation diagnosis across various anticoagulants.

The overall initiation of OAC treatment increased steadily in both patients with and without GIB (Fig. 3). The temporal trends in use of direct oral anticoagulants (DOACs) were similar, but a reduced dose of DOAC was more common among patients with GIB compared to those without GIB (33.2% vs. 22.6% of DOAC initiations). The rate of OAC initiation was consistently lower in patients with prior GIB compared to patients without GIB, averaging 52.9% and 62.5%, respectively. Among patients with GIBTAF, the rate of OAC initiation increased modestly but was significantly lower overall, averaging 34.1%.

Short‐term (90‐day) mortality after AF diagnosis was substantial and particularly pronounced among patients with GIBTAF (Table 2). The mortality rate was 5.6% in patients without GIB, compared to 11.5% in those with GIB. As death constitutes a competing event for treatment initiation, the considerable difference in mortality largely—but not entirely—accounts for the lower rate of OAC initiation among patients with GIB (Table S9). Mortality decreased slightly in all patient groups over the observation period (Fig. S2). Among patients who were not initiated on OAC, 90‐day mortality was markedly higher compared to those who received treatment. Mortality was comparable across anatomical and temporal GIB subgroups.

**Table 2:** Mortality within 90 days among all patients and those not receiving oral anticoagulation after new‐onset atrial fibrillation, stratified by gastrointestinal bleeding status, timing, and anatomical site.

Group	All patients (deaths/total *n*) (%)	No OAC (deaths/total *n*) (%)	OAC treatment (deaths/total *n*) (%)
No prior GIB	6247/111 369 (5.6)	5386/41 747 (12.9)	861/69 622 (1.2)
GIB	764/6628 (11.5)	676/3367 (20.1)	88/3261 (2.7)
**GIB subgroup**			
Upper	276/2610 (10.6)	239/1280 (18.7)	37/1330 (2.8)
Lower	66/669 (9.9)	57/301 (18.9)	9/368 (2.4)
Obscure	422/3349 (12.6)	380/1786 (21.3)	42/1563 (2.7)
**Time between GIB and AF diagnosis**			
GIB temporally with AF (±30 days)	204/1292 (15.8)	183/852 (21.5)	21/440 (4.8)
Prior GIB (>30 days)	560/5336 (10.5)	493/2515 (19.6)	67/2821 (2.4)

Abbreviations: AF, atrial fibrillation; GIB, gastrointestinal bleeding; OAC, oral anticoagulation.

In the entire study cohort, OAC was prescribed more often in patients over 65 years and in patients with hyperlipidemia (Table 3). Conversely, the likelihood of OAC initiation was lower in patients with dementia, abnormal liver function, alcohol abuse disorder, and current cancer, especially in patients with upper GI tract cancer. Anemia, thrombocytopenia, and chronic kidney disease were also associated with lower OAC initiation. Patients with GIB had a 16% lower likelihood of OAC initiation. Sensitivity analysis excluding patients with GIB did not significantly change the results (Table S10).

**Table 3:** Factors associated with oral anticoagulation treatment after new‐onset atrial fibrillation.

Variable	Valid *n*	Number of cases	Adjusted RR (95% CI)
**Age, years**			
Over 75 years	51 606	32 247	1.39 (1.36–1.42)
65–75 years	27 793	18 705	1.38 (1.36–1.41)
Under 65 years	13 145	6358	Reference
**Sex**			
Male	39 067	24 767	1.07 (1.06–1.08)
Female	53 477	32 543	Reference
**Annual income** ^a^			
Quartile 4 (highest)	22 296	13 702	1.04 (1.02–1.06)
Quartile 3	22 977	14 588	1.07 (1.05–1.08)
Quartile 2	22 320	14 382	1.08 (1.06–1.09)
Quartile 1 (lowest)	24 951	14 638	Reference
**Comorbidities**			
Hypertension	76 654	48 277	1.06 (1.04–1.07)
Hyperlipidemia	52 939	34 829	1.13 (1.12–1.14)
Diabetes	25 104	16 253	1.06 (1.05–1.07)
Coronary heart disease	25 382	15 528	0.95 (0.93–0.96)
Congestive heart failure	18 871	11 324	1.03 (1.02–1.04)
Ischemic stroke or TIA	17 903	11 013	0.97 (0.96–0.99)
Venous thromboembolism	7523	4617	1.02 (1.00–1.04)
Dementia	5969	2599	0.70 (0.68–0.72)
Abnormal liver function	598	207	0.70 (0.63 ‐ 0.78)
Psychiatric disease	14 605	8104	0.99 (0.97–1.00)
Alcohol abuse	3675	1657	0.79 (0.76–0.82)
Gastrointestinal bleeding	5293	2574	0.84 (0.81–0.86)
**Medication**			
Antiplatelet or NSAID	28 879	18 250	1.03 (1.02–1.04)
SSRI	5630	3087	0.94 (0.92–0.96)
PPI	51 968	31 748	1.00 (0.99–1.01)
**Cancer status**			
Current cancer	4861	1962	0.67 (0.64–0.69)
Previous cancer	18 712	11 572	0.97 (0.95–0.98)
Lower GI tract cancer	1752	946	0.99 (0.95–1.03)
Upper GI tract cancer	334	122	0.74 (0.65–0.85)
**Hemoglobin level** ^b^			
Anemia	25 199	13 264	0.84 (0.83–0.85)
Normal hemoglobin	67 345	44 046	Reference
**Platelet count** (mcL)^c^			
Under 100	1541	530	0.65 (0.61–0.70)
Over 100	91 003	56 780	Reference
**eGFR** (mL/min/1.73 m^2^)^d^			
<15	954	322	0.60 (0.55–0.65)
15–29	2621	1176	0.76 (0.72–0.79)
30–59	23 519	14 629	0.98 (0.97–0.99)
>60	65 450	41 183	Reference

*Note*: Patients with missing data were excluded from the regression analysis (*n* = 92 544).

Abbreviations: AF, atrial fibrillation; CI, confidence interval; eGFR, estimated glomerular filtration rate; GI, gastrointestinal; NSAID, nonsteroidal anti‐inflammatory drug; PPI, proton‐pump inhibitor; RR, relative risk; SSRI, selective serotonin reuptake inhibitor; TIA, transient ischemic attack.

^a^ Data were missing for 0.01% (*n* = 12).

^b^ Data were missing for 19.2% (*n* = 22 675).

^c^ Data were missing for 19.2% (*n* = 22 706).

^d^ Data were missing for 20.9% (*n* = 24 623).

Regarding the initiation of OAC therapy in patients with GIB, we found no differences between the UGIB and LGIB groups (Table 4). However, patients with OGIB exhibited the lowest probability of OAC initiation. Patients with prior GIB also demonstrated a 28% higher likelihood of OAC initiation compared to patients with GIBTAF. Sensitivity analyses yielded similar results when using narrower definitions of GIB (excluding ICD‐10 codes D50.0 and D62) and when excluding post‐AF GIB events to address potential reverse causation (Tables S11 and S12). The notable changes observed in the third sensitivity analysis, which eliminated the attributes of GIB (subgroup, temporal proximity), were the negative associations of heart disease, previous cancer, and upper GI tract cancer with OAC initiation (Table S13).

**Table 4:** Factors associated with oral anticoagulation treatment after new‐onset atrial fibrillation in patients with gastrointestinal bleeding.

Variable	Valid *n*	Number of cases	Adjusted RR (95% CI)
**Age, years**			
Over 75 years	3461	1712	1.38 (1.22–1.56)
65–75 years	1379	694	1.30 (1.14–1.47)
Under 65 years	453	168	Reference
**Sex**			
Male	2583	1254	1.08 (1.02–1.14)
Female	2710	1320	Reference
**Annual income** ^a^			
Quartile 4 (highest)	1296	659	1.12 (1.03–1.21)
Quartile 3	1254	642	1.10 (1.02–1.19)
Quartile 2	1226	601	1.09 (1.01–1.18)
Quartile 1 (lowest)	1517	672	Reference
**Comorbidities**			
Hypertension	4597	2273	1.06 (0.97–1.16)
Hyperlipidemia	3188	1690	1.20 (1.13–1.27)
Diabetes	1707	847	1.06 (1.01–1.13)
Coronary heart disease	1995	979	0.96 (0.90–1.02)
Congestive heart failure	1603	719	0.97 (0.91–1.03)
Ischemic stroke or TIA	1246	591	0.92 (0.86–0.98)
Venous thromboembolism	614	274	0.94 (0.86–1.03)
Dementia	479	156	0.65 (0.57–0.74)
Abnormal liver function	168	34	0.56 (0.41–0.75)
Psychiatric disease	1189	501	1.03 (0.95–1.11)
Alcohol abuse	496	160	0.70 (0.60–0.80)
**Medication**			
Antiplatelet or NSAID	1615	833	1.07 (1.01–1.14)
SSRI	388	167	0.96 (0.86–1.08)
PPI	4392	2185	1.06 (0.98–1.15)
**Cancer status**			
Current cancer	408	137	0.71 (0.62–0.82)
Previous cancer	1360	667	0.97 (0.91–1.03)
Lower GI tract cancer	268	128	1.04 (0.91–1.18)
Upper GI tract cancer	79	26	0.75 (0.55–1.02)
**Hemoglobin level** ^b^			
Anemia	2551	978	0.73 (0.69–0.77)
Normal hemoglobin	2742	1596	Reference
**Platelet count** (mcL)^c^			
Under 100	178	30	0.48 (0.35–0.67)
Over 100	5115	2544	Reference
**eGFR** (mL/min/1.73 m^2^)^d^			
<15	120	26	0.51 (0.37–0.71)
15–29	306	103	0.72 (0.61–0.84)
30–59	1655	809	0.97 (0.91–1.03)
>60	3212	1636	Reference
**GIB subgroup**			
Lower	519	283	1.00 (0.91–1.09)
Obscure	2695	1239	0.93 (0.88–0.99)
Upper	2079	1052	Reference
**Time between GIB and AF diagnosis**			
GIB temporally with AF (±30 days)	1037	348	0.72 (0.66–0.79)
Prior GIB (>30 days)	4256	2226	Reference

*Note*: Patients with missing data were excluded from the regression analysis (*n* = 5293).

Abbreviations: AF, atrial fibrillation; CI, confidence interval; eGFR, estimated glomerular filtration rate; GIB, gastrointestinal bleeding; NSAID, nonsteroidal anti‐inflammatory drug; PPI, proton‐pump inhibitor; RR, relative risk; SSRI, selective serotonin reuptake inhibitor; TIA, transient ischemic attack.

^a^ Data were missing for 0.01% (*n* = 1).

^b^ Data were missing for 18.7% (n = 1240).

^c^ Data were missing for 18.7% (*n* = 1244).

^d^ Data was missing for 19.7% (*n* = 1309).

## Discussion

The main findings of this study were: (1) Initiation of OAC was less likely in patients with GIB, particularly among patients with GIBTAF or OGIB. (2) The use of OACs increased steadily in both patients with and without GIB from 2010 to 2018 but remained less utilized in patients with GIB. (3) Compared to patients without GIB, patients with GIB were older, more often male, and had a higher comorbidity burden.

In our study, 5.6% of patients with new‐onset AF (mean age 75.5 years) had a history of prior GIB or GIB concurrently with AF diagnosis. A previous cohort study conducted in the general population in Finland among 39 054 participants from 1987 to 2016, with a median follow‐up duration of 14.9 years, reported that 2.8% of the participants experienced major GIB, with a crude incidence rate of 174.0 per 100 000 person‐years [15]. A systematic review of the global epidemiology of GIB reported varying incidence rates ranging from 15.0 to 172.0 per 100 000 person‐years for UGIB and from 20.5 to 87.0 per 100 000 person‐years for LGIB [10]. The high incidence of GIB among AF patients in our study before the initiation of OAC therapy was not unexpected, considering the overlapping risk factors between GIB and AF, such as advanced age [16], multimorbidity [17], and alcohol abuse [18].

Research on the outcomes of OAC therapy choices in patients with GIB predominantly have involved those who were on OAC therapy at the time of GIB. Resumption of OAC after GIB has been associated with a significant reduction in all‐cause mortality and thromboembolic events but also with an increased risk of recurrent GIB [19, 20]. Because of the favorable risk–benefit ratio, prompt resumption of OAC after GIB has been recommended in patients who have been on OAC therapy [4]. Studies on the impact of GIB on the initiation of OAC therapy in patients with new‐onset AF are limited, but prior GIB has not been associated with an increased risk of rebleeding [11]. Because the causes of GIB are similar regardless of OAC exposure [21], it is plausible that the approach to reinitiate OAC therapy after GIB also applies when initiating OAC therapy at the time of AF onset.

The assessment of individual bleeding risk related to OAC therapy is multifactorial and should not be viewed as a static, one‐time evaluation. Instead, bleeding risk is dynamic, evolving over time due to factors such as aging, newly diagnosed comorbidities, and changes in medication [22]. In our study, the observed lower initiation rate of OAC therapy in patients with GIB should not be solely attributed to the GIB. High age and comorbidities (especially dementia, alcohol abuse, and cancer), along with lower hemoglobin levels and eGFR, reflect the overall frailty of these patients, which predisposes them to bleeding complications. Consequently, these patient‐related factors, other than GIB, might explain the lower likelihood of OAC initiation in patients with GIB.

Importantly, many of these same factors are also associated with increased mortality, as observed in patients with GIB in our study. It is therefore possible that frailty might have been a major determinant in the decision not to initiate OAC therapy in these high‐risk patients [23]. The substantially higher short‐term mortality observed in patients who were not initiated on OAC should not be interpreted as evidence of harm from undertreatment. Rather, it may reflect a clinical decision to avoid futile and potentially harmful anticoagulation in cases where life expectancy was judged to be limited [24].

Unlike many other risk factors for bleeding, the risk of recurrent GIB is modifiable if the source of bleeding is identified and endoscopic hemostasis is applied when necessary [25]. This underscores the importance of a structured, dynamic reassessment of bleeding risk over time, along with targeted correction of modifiable risk factors, to ensure that patients who could benefit from anticoagulation are not inappropriately denied treatment [22]. In cases where OAC therapy cannot be used, left atrial appendage closure devices may be considered an alternative treatment strategy [26].

Because numerous etiologies of GIB share the same clinical manifestations, endoscopic examination is the primary method to identify the bleeding site. There are various reasons for abstaining from endoscopic examination, including less severe clinical manifestations of GIB, an unfavorable benefit‐to‐risk ratio for endoscopy, or decisions regarding limitations of care [27, 28]. The reasons for the infrequent use of endoscopic examinations (1.8%) and the unidentified etiology of GIB in patients categorized as OGIB in our study are speculative. Nonetheless, high age, low annual income, and comorbidities suggest that these patients could be more fragile than other patients with GIB, and thus endoscopic examinations were not performed. These same patient‐related factors might also explain the lower likelihood of OAC initiation among patients with OGIB. Additionally, their GIB was temporally closer to the AF onset than in UGIB and LGIB patients.

The steady increase in the initiation of OAC therapy from 2010 to 2018 in our study is consistent with findings from other countries and represents a continuation of a trend that began earlier in the 2000s [29]. A major factor in the underuse of OACs is the concern over bleeding complications, and consequently, episodes of GIB might reduce OAC initiation [30]. The risk of recurrent GIB varies significantly, with the severity of the initial GIB being a primary concern [25, 31]. We did not observe differences in OAC initiation when comparing patients with UGIB and LGIB. In our study, the temporal proximity of GIB significantly reduced the likelihood of OAC initiation and the utilization of OACs increased only modestly in patients with GIBTAF. Our observation period covers the introduction of DOACs, and the only difference in anticoagulant usage between patients with or without GIB was the higher proportion of reduced DOAC dose among those with GIB.

Our study has many strengths, the most notable being the comprehensive nationwide population‐based cohort with accompanying laboratory data. We employed several overlapping registries of high validity to define new‐onset AF and relevant comorbidities [32]. The hospital registry data have been well validated for identifying GIB, with improved accuracy in anatomical site categorization through the incorporation of secondary diagnosis codes and endoscopic procedures [33, 34]. Nonetheless, limitations inherent to registry studies also apply to our study. Specific details of GIB episodes, including bleeding severity and the exact cause of bleeding, are not directly collected in Finnish hospital registries. Thus, the etiologies of GIB are assumptions based on registry data and prior knowledge of its use in epidemiological research. Additionally, our study included only the GIB episode closest to AF onset, ignoring the possible relevance of recurrent GIB episodes. The inclusion of diagnosis codes D50.0 and D62.0, even with an extended list of exclusion criteria to rule out concurrent extraintestinal bleeding, might still have led to the misclassification of bleeding events. Due to potential date disparities across registers, patients with GIBTAF (±30 days of AF diagnosis) were included in the GIB cohort. Therefore, it is possible that GIB episodes occurring after AF onset under OAC exposure were included. Data of aspirin use were unavailable due to its over‐the‐counter status in Finland, as well as the smoking status of the patients. Our findings predominantly apply to White Europeans and may not be directly applicable to other populations.

In conclusion, a significant proportion of patients with new‐onset AF presented with a history of GIB. GIB patients are typically older and have a high prevalence of comorbidities. Despite the increasing use of OACs, they remain less utilized in patients with GIB. Initiation of OAC is more likely if the cause of GIB has been identified and when more time has elapsed between the GIB episode and AF onset. Further research is needed to evaluate the outcomes of patients with new‐onset AF and GIB in relation to the choice of OAC therapy.

## Author contributions

**Santeri Jolkkonen**: Conceptualization; methodology; investigation; data curation; formal analysis; writing—original draft; and visualization and directly accessed and verified the underlying data reported in the article. **Jukka Putaala**: Conceptualization; methodology; writing—review and editing; supervision. **Konsta Teppo**: Conceptualization; methodology; data curation; writing—review and editing. **Pirjo Mustonen**: Conceptualization; methodology; writing—review and editing; supervision. **Jussi Jaakkola**: Conceptualization; methodology; writing—review and editing. **Olli Halminen**: Conceptualization; methodology; data curation; investigation; writing—review and editing; project administration. **Ossi Lehtonen**: Conceptualization; methodology; writing—review and editing. **Jari Haukka**: Conceptualization; methodology; data curation; writing—review and editing. **Miika Linna**: Conceptualization; methodology; writing—review and editing. **Aapo Aro**: Conceptualization; methodology; writing—review and editing. **Juha Hartikainen**: Conceptualization; methodology; writing—review and editing. **Juhani Airaksinen**: Conceptualization; methodology; writing—review and editing; project administration. **Mika Lehto**: Conceptualization; methodology; writing—review and editing; supervision; project administration; funding acquisition.

## Conflict of interest statement

Santeri Jolkkonen: research grants: Pulsus Foundation, and The Foundation of Medical Licentiate Paavo Ilmari Ahvenainen. Jukka Putaala: speaker: Bayer, Boehringer Ingelheim, BMS‐Pfizer, Abbott; Advisory board: Novo Nordisk, and Herantis Pharma; visiting editor: Terve Media; and stock ownership: VitalSignum. Konsta Teppo: research grants: the Finnish Foundation for Cardiovascular Research, Aarne and Aili Turunen Foundation, the Finnish Medical Foundation, the Finnish Foundation for Alcohol Studies, and the Finnish State Research Funding. Pirjo Mustonen: consultant: Roche, BMS‐Pfizer‐alliance, Novartis Finland, Boehringer Ingelheim, and MSD Finland. Jussi Jaakkola: none declared. Aapo Aro: research grants: Finnish Foundation for Cardiovascular Research, and Sigrid Juselius Foundation; and speaker: Abbott, Johnson & Johnson, Sanofi, Bayer, and Boehringer‐Ingelheim. Olli Halminen: none declared. Ossi Lehtonen: none declared. Jari Haukka: consultant: Research Janssen R&D; and speaker: Bayer Finland. Miika Linna: speaker: BMS‐Pfizer‐alliance, Bayer, and Boehringer Ingelheim. Jari Haukka: research grants: the Finnish Foundation for Cardiovascular Research, EU Horizon 2020, and EU FP7; advisory board member: BMS‐Pfizer‐alliance, Novo Nordisk, and Amgen; and speaker: Cardiome, and Bayer. K. E. Juhani Airaksinen: research grants: the Finnish Foundation for Cardiovascular Research; speaker: Bayer, Pfizer, and Boehringer Ingelheim. Mika Lehto: consultant: BMS‐Pfizer‐alliance, Bayer, Boehringer Ingelheim, and MSD; speaker: BMS‐Pfizer‐ alliance, Bayer, Boehringer Ingelheim, MSD, Terve Media, and Orion Pharma; research grants: Aarne Koskelo Foundation, the Finnish Foundation for Cardiovascular Research, and Helsinki and Uusimaa Hospital District research fund.

## Funding information

The FinACAF project is supported by Helsinki and Uusimaa Hospital District research fund (grant numbers TYH2019309, TYH2023319); The Finnish Foundation for Cardiovascular Research; Aarne Koskelo Foundation; Yrjö Jahnsson Foundation; and Sigrid Juselius Foundation. This work was supported by the Pulsus Foundation, and the Foundation of Medical Licentiate Paavo Ilmari Ahvenainen. The funders had no role in the design and conduct of the study; collection, management, analysis, and interpretation of the data; preparation, review, or approval of the manuscript; and decision to submit the manuscript for publication.

## Previous presentation

These data were presented in part as a poster at the European Heart Rhythm Association (EHRA) 2022 Congress, April 3–5, 2022, in Copenhagen, Denmark.

## Supporting information




**Supplementary Table 1**: Definitions and further details of the baseline characteristics.
**Supplementary Table 2**: List of ICD‐10 and NCSP codes for gastrointestinal bleeding categorization alongside with the summary of categorization process.
**Supplementary Table 3**: Summary of ICD‐10 code counts for Upper gastrointestinal bleeding group.
**Supplementary Table 4**: Summary of ICD‐10 code counts for Lower gastrointestinal bleeding group.
**Supplementary Table 5**: Summary of ICD‐10 code counts for Obscure gastrointestinal bleeding group.
**Supplementary Table 6**: Summary of NCSP code counts for Upper gastrointestinal bleeding group.
**Supplementary Table 7**: Summary of NCSP code counts for Lower gastrointestinal bleeding group.
**Supplementary Table 8**: Summary of NCSP code counts for Obscure gastrointestinal bleeding group.
**Supplementary Table 9**: Incidence of oral anticoagulation initiation and competing risk of death within 90 days after atrial fibrillation diagnosis, stratified by gastrointestinal bleeding status, with subdistribution hazard ratios (Fine‐Gray model).
**Supplementary Table 10**: Factors associated with oral anticoagulation initiation among patients without prior gastrointestinal bleeding.
**Supplementary Table 11**: Factors associated with oral anticoagulation initiation among patients with gastrointestinal bleeding (GIB), excluding ICD‐10 codes D50.0 and D62 from the GIB definition.
**Supplementary Table 12**: Factors associated with oral anticoagulation initiation among patients with gastrointestinal bleeding (GIB), excluding those who experienced GIB after the diagnosis of new‐onset atrial fibrillation.
**Supplementary Table 13**: Factors associated with oral anticoagulation initiation among patients with prior gastrointestinal bleeding after eliminating attributes of GIB (subgroup, temporal proximity).
**Supplementary Table 14**: Summary of the time gap between baseline laboratory testing date and cohort entry date.
**Supplementary Table 15**: Comparison of baseline characteristics between patients included in the regression analysis and those excluded due to missing data.

**STROBE Statement**—Checklist of items that should be included in reports of cohort studies.

## Data Availability

Given the sensitive character of the data compiled for this study, requests for dataset access from qualified researchers with expertise in human subject confidentiality protocols can be forwarded to the Finnish national register holders (KELA, Finnish Institute for Health and Welfare, Population Register Center, and Tax Register) via Findata (https://findata.fi/en/).
