# Supplementary material for: Oral anticoagulation in patients with gastrointestinal bleeding and new‐onset atrial fibrillation: A population‐based registry‐linkage study
Source: J Intern Med. 2025 Sep 23;298(5):450–63. doi: 10.1111/joim.70018 (PMC12522523; doi:10.1111/joim.70018)
Supplement: Supplementary file 1 — Supplementary Table 1: Definitions and further details of the baseline characteristics. Supplementary Table 2: List of ICD‐10 and NCSP codes for gastrointestinal bleeding categorization alongside with the summary of categorization process. Supplementary Table 3: Summary of ICD‐10 code counts for Upper gastrointestinal bleeding group. Supplementary Table 4: Summary of ICD‐10 code counts for Lower gastrointestinal bleeding group. Supplementary Table 5: Summary of ICD‐10 code counts for Obscure gastrointestinal bleeding group. Supplementary Table 6: Summary of NCSP code counts for Upper gastrointestinal bleeding group. Supplementary Table 7: Summary of NCSP code counts for Lower gastrointestinal bleeding group. Supplementary Table 8: Summary of NCSP code counts for Obscure gastrointestinal bleeding group. Supplementary Table 9: Incidence of oral anticoagulation initiation and competing risk of death within 90 days after atrial fibrillation diagnosis, stratified by gastrointestinal bleeding status, with subdistribution hazard ratios (Fine‐Gray model). Supplementary Table 10: Factors associated with oral anticoagulation initiation among patients without prior gastrointestinal bleeding. Supplementary Table 11: Factors associated with oral anticoagulation initiation among patients with gastrointestinal bleeding (GIB), excluding ICD‐10 codes D50.0 and D62 from the GIB definition. Supplementary Table 12: Factors associated with oral anticoagulation initiation among patients with gastrointestinal bleeding (GIB), excluding those who experienced GIB after the diagnosis of new‐onset atrial fibrillation. Supplementary Table 13: Factors associated with oral anticoagulation initiation among patients with prior gastrointestinal bleeding after eliminating attributes of GIB (subgroup, temporal proximity). Supplementary Table 14: Summary of the time gap between baseline laboratory testing date and cohort entry date. Supplementary Table 15: Comparison of baseline characteristics between [file JOIM-298-450-s002.docx]

**Oral anticoagulation in patients with gastrointestinal bleeding and
new-onset atrial fibrillation: A population-based registry-linkage study**

Santeri Jolkkonen, MD^1,2^, Jukka Putaala, MD, PhD^3^, Konsta Teppo, MD, PhD^4^, Pirjo Mustonen, MD, PhD^4^, Jussi Jaakkola, MD, PhD^4,5^, Aapo Aro, MD, PhD^6^, Olli Halminen, MSc, PhD^7^, Ossi Lehtonen, MSc^8^, Jari Haukka, PhD, Adj. Prof.^9^, Miika Linna, PhD, Prof.^8^, Juha Hartikainen, MD, PhD, Prof.^10^, K.E. Juhani Airaksinen, MD, PhD, Prof.^4^, Mika Lehto, MD, PhD^11^

^1^ Faculty of Medicine, University of Helsinki, Helsinki, Finland

^2^ Department of Gastroenterology and Alimentary Tract Surgery, Tampere University Hospital, Tampere, Finland

^3^ Department of Neurology, HUS Helsinki University Hospital and University of Helsinki, Helsinki, Finland

^4^ Heart Center, Turku University Hospital and University of Turku, Turku, Finland

^5^ Heart Unit, Satasairaala, Pori, Finland

^6^ Heart and Lung Center, HUS Helsinki University Hospital and University of Helsinki, Helsinki, Finland

^7^ Department of Industrial Engineering and Management, Aalto University, Espoo, Finland

^8^ Department of Health and Social Management, University of Eastern Finland, Kuopio, Finland

^9^ Department of Public Health, Clinicum, University of Helsinki, Helsinki, Finland

^10^ Heart Center, Kuopio University Hospital and University of Eastern Finland, Kuopio, Finland

^11^ Department of Internal Medicine, Jorvi Hospital, HUS Helsinki University Hospital, Espoo, Finland; and University of Helsinki, Helsinki, Finland

**TABLE OF CONTENTS**

**Supplementary Figure 1.** Flowchart of FinACAF incidence cohort with laboratory data and indication for long-term oral anticoagulation treatment.

**Supplementary Figure 2.** Temporal trends in 90-day mortality after atrial fibrillation (AF) diagnosis from 2010 to 2018, stratified by gastrointestinal bleeding status, timing, and site.

**Supplementary Table 1.** Definitions and further details of the baseline characteristics

**Supplementary Table 2.** List of ICD-10 and NCSP codes for gastrointestinal bleeding categorization alongside with the summary of categorization process

**Supplementary Table 3.** Summary of ICD-10 code counts for Upper gastrointestinal bleeding group

**Supplementary Table 4.** Summary of ICD-10 code counts for Lower gastrointestinal bleeding group

**Supplementary Table 5.** Summary of ICD-10 code counts for Obscure gastrointestinal bleeding group

**Supplementary Table 6.** Summary of NCSP code counts for Upper gastrointestinal bleeding group

**Supplementary Table 7.** Summary of NCSP code counts for Lower gastrointestinal bleeding group

**Supplementary Table 8.** Summary of NCSP code counts for Obscure gastrointestinal bleeding group

**Supplementary Table 9.** Incidence of oral anticoagulation initiation and competing risk of death within 90 days after atrial fibrillation diagnosis, stratified by gastrointestinal bleeding status, with subdistribution hazard ratios (Fine-Gray model)

**Supplementary Table 10.** Factors associated with oral anticoagulation initiation among patients without prior gastrointestinal bleeding

**Supplementary Table 11.** Factors associated with oral anticoagulation initiation among patients with gastrointestinal bleeding (GIB), excluding ICD-10 codes D50.0 and D62 from the GIB definition

**Supplementary Table 12.** Factors associated with oral anticoagulation initiation among patients with gastrointestinal bleeding (GIB), excluding those who experienced GIB after the diagnosis of new-onset atrial fibrillation

**Supplementary Table 13.** Factors associated with oral anticoagulation initiation among patients with prior gastrointestinal bleeding after eliminating attributes of GIB (subgroup, temporal proximity)

**Supplementary Table 14.** Summary of the time gap between baseline laboratory testing date and cohort entry date

**Supplementary Table 15.** Comparison of baseline characteristics between patients included in the regression analysis and those excluded due to missing data

**Supplementary references**

**Supplementary Figure 1.** Flowchart of FinACAF cohort with laboratory data and definite indication for long-term oral anticoagulation treatment.

Abbreviations: FinACAF, The Finnish AntiCoagulation in Atrial Fibrillation Study; ICD-10, The International Classification of Diseases, Tenth Revision; AF; atrial fibrillation, OAC, oral anticoagulation; CHA_2_DS_2_-VASc, congestive heart failure, hypertension, age ≥75 years, diabetes, history of stroke or TIA, vascular disease, age 65–74 years, sex category (female).

**Supplementary Figure 2.** Temporal trends in 90-day mortality after atrial fibrillation diagnosis from 2010 to 2018, stratified by gastrointestinal bleeding status, timing, and site.

Abbreviations: AF; atrial fibrillation, GIBTAF; gastrointestinal bleeding temporally with atrial fibrillation, GIB; gastrointestinal bleeding.

**Supplementary Table 1.** Definitions and further details of the baseline characteristics

|  | ICD-10 | ICPC-2 | Reimbursement code | ATC code | Other |
| --- | --- | --- | --- | --- | --- |
| Annual taxable income^1^ |  |  |  |  | National Tax Register |
| Hypertension | I10-I15 | K85, K86, K87 | 205 | C03A, C03B, C03DB, C03EA, C07A, C08CA, C08D, C09 |  |
| Hyperlipidemia | E78 | T93 | 206 | C10 |  |
| Diabetes | E10-E14 | T89, T90 | 103, 215 | A10 |  |
| Coronary heart disease | I21-I25 |  |  |  |  |
| Congestive heart failure | I50, I11.0, I13.0, I13.2 | K77 | 201 |  |  |
| Ischemic stroke or TIA | I63, I64, I69.3-I69.8, G45 | K89, K90 |  |  |  |
| Venous thromboembolism | I26, I80-183 |  |  |  |  |
| Dementia | F00-F03, G30 |  |  |  |  |
| Abnormal liver function | K70.2-K70.4, K71.7, K71.8, K72, K74 |  |  |  |  |
| Psychiatric disorder | F04-F99 |  |  |  |  |
| Alcohol abuse | F10 |  |  |  |  |
| Antiplatelets or NSAIDs^2^ |  |  |  | B01AC, M01A |  |
| SSRIs^2^ |  |  |  | N06AB |  |
| PPIs^2^ |  |  |  | A02BC |  |
| Cancer^3^ |  |  |  |  | Any cancer registered in the Finnish Cancer Registry |
| GI tract cancer^4^ | C15-C21, D01.0-D01.1 |  |  |  |  |

Abbreviations: ICD-10, International Classification of Diseases, Tenth Revision; ICPC-2, International Classification of Primary Care, Second Edition; ATC, anatomic therapeutic chemical; TIA, transient ischemic attack; NSAID, nonsteroidal anti-inflammatory drug; SSRI, selective serotonin reuptake inhibitor; PPI, Proton-pump inhibitor; GI, gastrointestinal.

^1^The patient’s highest annual taxable income (in 1000-euro accuracy) during the FinACAF study’s observation period 2004–2018 was obtained from the national Tax Register. The annual income was capped to a maximum of 100 000 euros to avoid patients’ identifiability due to high incomes.

^2^History of medication use was defined as medication purchases by the medication group from the year preceding cohort entry, excluding purchases from the most recent month.

^3^Cancer history was gathered from The Finnish Cancer Registry from 2004 until cohort entry. Cancer was defined as active if the patient received a diagnosis within the year prior to cohort entry or received chemotherapy or radiation therapy within the year prior to cohort entry. Otherwise, cancer history was defined as previous.

^4^Luminal gastrointestinal tract cancers were classified into Upper or Lower GI tract cancer categories based on their location relative to the ligament of Treitz.

**Supplementary Table 2.** List of ICD-10 and NCSP codes for gastrointestinal bleeding (GIB) categorization alongside with the summary of categorization process

**ICD-10 codes for gastrointestinal bleeding**

| 1. Upper GIB | I850, K221, K223, K226, K250, K252, K254, K256, K260, K262,K264, K266, K270, K272, K274, K276, K290 |
| --- | --- |
| 2. Lower GIB | K280, K282, K284, K286 |
| 3. Unspecific GIB | K920, K921, K922, K625 |
| 4. Anemia due to acute blood loss | D500, D62 |

**Accessory ICD-10 codes**

| 5. Upper GI tract | I859, K210, K228, K229, K251, K253, K255, K257, K259, K261, K263, K265, K267, K269, K271, K273, K275, K277, K279, K281, K283, K285, K287, K289, K291, K292, K293, K295, K296, K297, K298, K299, C150, C151, C152, C153, C154, C155, C158, C159, C160, C1600, C1601, C1602, C1603, C1604, C1609, C161, C1610, C1611, C1612, C1613, C1614, C1619, C162, C1620, C1621, C1622, C1623, C1624, C1629, C163, C1630, C1631, C1632, C1633, C1634, C1639, C164, C165, C166, C168, C169, C170, D001, D002, D130, D131, D132, D371 |
| --- | --- |
| 6 Lower GI tract | K570, K571, K572, K573, K574, K575, K578, K579, K552, K600, K601, K602, K603, K604, K605, K62, K620, K621, K622, K623, K626, K627, K628, K629, K640, K641, K642, K643, K644, K645, K648, K649, K500, K501, K508, K509, K510, K511, K512, K513, K514, K515, K518, K519, C171, C172, C173, C178, C179, C180, C1800, C1801, C1802, C1809, C181, C1810, C1811, C1812, C1819, C182, C1820, C1821, C1822, C1829, C183, C1830, C1831, C1832, C1839, C184, C1840, C1841, C1842, C1849, C185, C1850, C1851, C1852, C1859, C186, C1860, C1861, C1862, C1869, C187, C1870, C1871, C1872, C1879, C188, C1880, C1881, C1882, C1889, C189, C1890, C1891, C1899, D010, D011, C19, C2090, C2091, C2092, C2099, C210, C211, C212, C218, D012, D013, D120, D121, D122, D123, D124, D125, D126, D127, D128, D129, D133, D372, D373, D374, D375, K635 |
| 7. Intermediate GI tract | K631, K633, K558, K550, K551, K558, K559 |

**NCSP codes for GI tract endoscopic interventions and procedures**

| 8. Upper GI tract | JCA20, JCA22, JCA32, JCA35, JCA38, JCA42, TJC00, JDA22, JDA32, JDA35, JDA42, JDA52, JDA60, JDA61, UJD02, UJD10 |
| --- | --- |
| 9. Lower GI tract | JFA22, JFA25, JFA28, JFA32, JFA35, JFA42, JFA45, JFA48, JFA52, JFA55, JGA22, JGA28, JGA32, JGA35, UJF02, UJF12, UJF14, UJF22, UJF30, UJF32, UJF34, UJF45, UJF92, UJG02 |

**ICD-10 codes for exclusion criteria**

| 10. Other bleeding source outside of the GI tract | J942, N02, R31, R040, R041, R042, R048, R049, R58, N938, N939, H922, D683, I600, I601, I602, I603, I604, I605, I606, I607, I608, I609, I610, I611, I612, I613, I614, I615, I616, I618, I619, I620, I621, I629, I690, I691, I692, S062, S063 |
| --- | --- |
| 11. Major trauma or complication of intervention | S02, S020, S0200, S0201, S021, S0210, S0211, S022, S0220, S0221, S023, S0230, S0231, S024, S0240, S0241, S0247, S026, S0260, S0261, S0262, S0263, S0264, S0265, S0266, S0267, S0269, S027, S0270, S0271, S028, S0280, S0281, S029, S0290, S0291, S06, S061, S062, S063, S064, S065, S066, S067, S068, S069, S07, S070, S071, S078, S079, S08, S080, S088, S089, S090, S110, S111, S112, S12, S120, S121, S122, S127, S128, S129, S131, S15, S150, S151, S152, S153, S157, S158, S159, S17, S170, S178, S179, S18, S22, S220, S221, S222, S223, S224, S225, S228, S229, S230, S231, S25, S250, S251, S252, S253, S254, S255, S257, S258, S259, S26, S260, S268, S269, S27, S270, S271, S272, S273, S274, S275, S276, S277, S278, S279, S28, S280, S281, S32, S320, S321, S322, S323, S324, S325, S327, S328, S330, S331, S332, S333, S334, S35, S350, S351, S352, S353, S354, S355, S357, S358, S359, S36, S360, S361, S362, S363, S364, S365, S366, S367, S368, S369, S37, S370, S371, S372, S373, S374, S375, S376, S377, S378, S379, S38, S380, S381, S382, S383, S396, S42, S420, S421, S422, S423, S424, S427, S428, S429, S45, S450, S451, S452, S457, S458, S459, S47, S48, S480, S481, S489, S52, S520, S521, S522, S523, S524, S525, S526, S527, S528, S529, S55, S550, S551, S552, S557, S558, S559, S57, S570, S578, S579, S58, S580, S581, S589, S62, S620, S621, S622, S623, S624, S628, S65, S650, S651, S652, S653, S657, S658, S659, S67, S678, S68, S683, S684, S688, S689, S72, S720, S721, S722, S723, S724, S727, S728, S729, S75, S750, S751, S752, S757, S758, S759, S77, S770, S771, S772, S78, S780, S781, S789, S82, S820, S821, S822, S823, S824, S825, S826, S827, S828, S829, S85, S850, S851, S852, S853, S854, S855, S857, S858, S859, S87, S870, S878, S88, S880, S881, S889, S92,S920, S921, S922, S923, S927, S929, S95, S950, S951, S952, S957, S958, S959, S97, S970, S978, S98, S980, S983, S984, T01, T02, T020, T021, T022, T023, T024, T025, T026, T027, T028, T029, T04, T040, T041, T042, T043, T044, T047, T048, T049, T05, T050, T051, T052, T053, T054, T055, T056, T058, T059, T063, T065, T068, T07, T08, T09, T096, T10, T114, T116, T12, T134, T136, T142, T145, T147, T27, T270, T271, T272, T273, T274, T275, T276, T277, T28, T280, T281, T282, T283, T284, T285, T286, T287, T288, T289, T29, T311, T312, T313, T314, T315, T316, T317, T318, T319, T321, T322, T323, T324, T325, T326, T327, T328, T329, T790, T791, T792, T794, T797, T798, T799, T80, T801, T81, T810, T811, T812, T813, T8158, T816, T817, T818, T819, T82, T820, T821, T822, T823, T824, T825, T828, T829, T83, T830, T831, T832, T833, T834, T838, T839, T84, T840, T841, T842, T843, T844, T848, T849, T85, T850, T851, T853, T854, T855, T856, T858, T859, T87, T870, T871, T872, T875, T876, T88, T882, T885, T888, T889 |

**Summary of the categorization process of gastrointestinal bleedings**

The last gastrointestinal bleeding (GIB) event in HILMO (hospitalizations and outpatient specialist visits register) before atrial fibrillation (AF) diagnosis was considered for analysis.

If upper GIB (1), lower GIB (2), or unspecific GIB (2) was present, the patient was assigned directly to prior GIB group. If anemia due to acute blood loss (3) was present, the patient was assigned to the prior GIB group after exclusion of other concurrent bleeding source outside of the GI tract (9), or major trauma or complication of intervention (10) [1,2].

Patients with prior GIB were categorized into three groups according to this algorithm:

**Upper gastrointestinal bleeding**

- Specific upper GIB (1) OR
- Unspecific GIB (3) and concurrent upper GI tract diagnosis (5) or endoscopic procedure (8) without concurrent lower GI tract endoscopic intervention (9) OR
- Intermediate GI tract diagnosis (7) and concurrent upper GI tract endoscopic procedure (8) without concurrent lower GI tract endoscopic procedure (9)

**Lower gastrointestinal bleeding**

- Specific lower GIB (2) OR
- Unspecific GIB (3) and concurrent lower GI tract diagnosis (6) or endoscopic procedure (9) without concurrent upper GI tract endoscopic intervention (8) OR
- Intermediate GI tract diagnosis (7) and concurrent lower GI tract endoscopic procedure (9) without concurrent upper GI tract endoscopic procedure (8)

**Obscure gastrointestinal bleeding**

- Unspecific GIB (3) or anemia due to acute blood loss (4) without concurrent GI tract diagnosis (1, 2, 5, 6) or endoscopic procedure (8, 9) OR
- Intermediate GI tract diagnosis (7) without concurrent endoscopic procedure (8, 9) OR
- If a clear classification could not be assigned

The result of the categorization process was checked manually if a patient was initially categorized into more than one group or if patients had multiple diagnoses or endoscopic procedural codes. The appropriate group was chosen based on the most clinically significant diagnosis or endoscopic procedural code. Altogether, in 101 (1.5%) cases, the GIB group was changed manually.

**Supplementary Table 3.** Summary of ICD-10 code counts for Upper GIB group

| **ICD10** | **Description** | **Count** |
| --- | --- | --- |
| Total |  | 3226 |
| K221 | Ulcer of esophagus | 680 |
| K921 | Melena | 341 |
| K250 | Acute gastric ulcer with hemorrhage | 285 |
| K254 | Chronic or unspecified gastric ulcer with hemorrhage | 241 |
| K260 | Acute duodenal ulcer with hemorrhage | 219 |
| K290 | Acute haemorrhagic gastritis | 161 |
| D500 | Iron deficiency anaemia secondary to blood loss | 154 |
| K920 | Hematemesis | 140 |
| K226 | Gastro-esophageal laceration-hemorrhage syndrome | 139 |
| K264 | Chronic or unspecified duodenal ulcer with hemorrhage | 107 |
| K210 | Gastro-oesophageal reflux disease with oesophagitis | 98 |
| K922 | Gastrointestinal hemorrhage, unspecified | 79 |
| I850 | Esophageal varices with bleeding | 49 |
| K253 | Acute gastric ulcer without hemorrhage or perforation | 46 |
| K259 | Gastric ulcer, unspecified as acute or chronic, without hemorrhage or perforation | 40 |
| K223 | Perforation of esophagus | 37 |
| K625 | Hemorrhage of anus and rectum | 33 |
| D62 | Acute posthaemorrhagic anaemia | 29 |
| K573 | Diverticular disease of large intestine without perforation and abscess | 26 |
| D131 | Benign neoplasm of stomach | 19 |
| K252 | Acute gastric ulcer with both hemorrhage and perforation | 18 |
| K257 | Chronic gastric ulcer without hemorrhage or perforation | 18 |
| K293 | Chronic superficial gastritis | 18 |
| K297 | Gastritis, unspecified | 18 |
| K263 | Acute duodenal ulcer without hemorrhage or perforation | 17 |
| K269 | Duodenal ulcer, unspecified as acute or chronic, without hemorrhage or perforation | 17 |
| K270 | Acute peptic ulcer, site unspecified, with hemorrhage | 15 |
| K295 | Unspecified chronic gastritis | 15 |
| K256 | Chronic or unspecified gastric ulcer with both hemorrhage and perforation | 14 |
| I859 | Esophageal varices without bleeding | 14 |
| K262 | Acute duodenal ulcer with both hemorrhage and perforation | 8 |
| K291 | Other acute gastritis | 6 |
| K298 | Duodenitis | 6 |
| K299 | Gastroduodenitis, unspecified | 6 |
| K274 | Chronic or unspecified peptic ulcer, site unspecified, with hemorrhage | 5 |
| K296 | Other gastritis | 5 |
| C155 | Cancer of lower third of esophagus | 5 |
| D132 | Benign neoplasm of duodenum | 5 |
| K558 | Other vascular disorders of intestine | 5 |
| K255 | Chronic or unspecified gastric ulcer with perforation | 4 |
| C1600 | Cancer of the cardia region without histological confirmation | 4 |
| D371 | Neoplasm of uncertain or unknown behavior of stomach | 4 |
| K266 | Chronic or unspecified duodenal ulcer with both hemorrhage and perforation | 3 |
| C159 | Unspecified cancer of esophagus | 3 |
| K519 | Ulcerative colitis, unspecified | 3 |
| D125 | Benign neoplasm of sigmoid colon | 3 |
| D126 | Benign neoplasm of unspecified part of colon | 3 |
| D127 | Benign neoplasm of rectosigmoid junction | 3 |
| D372 | Neoplasm of uncertain or unknown behavior of small intestine | 3 |
| K550 | Acute vascular disorder of intestine | 3 |
| K251 | Acute gastric ulcer with perforation | 2 |
| K267 | Chronic duodenal ulcer without hemorrhage or perforation | 2 |
| K279 | Peptic ulcer, site unspecified, unspecified as acute or chronic, without hemorrhage or perforation | 2 |
| C1623 | Cancer of the body of stomach, diffuse carcinoma | 2 |
| C1639 | Cancer of the pyloric orifice, other or unspecified carcinoma | 2 |
| K571 | Diverticular disease of small intestine without perforation and abscess | 2 |
| K508 | Other Crohn's disease | 2 |
| K509 | Crohn's disease, unspecified | 2 |
| D133 | Benign neoplasm of other or unspecified part of small intestine | 2 |
| K551 | Chronic vascular disorders of intestine | 2 |
| K272 | Acute peptic ulcer, site unspecified, with both hemorrhage and perforation | 1 |
| K228 | Other specified diseases of oesophagus | 1 |
| K229 | Disease of oesophagus, unspecified | 1 |
| K261 | Acute duodenal ulcer with perforation | 1 |
| K265 | Chronic or unspecified duodenal ulcer with perforation | 1 |
| C151 | Cancer of thoracic part of esophagus | 1 |
| C1609 | Cancer of the cardia, other or unspecified carcinoma | 1 |
| C1620 | Cancer of the body of stomach without histological confirmation | 1 |
| C1624 | Cancer of the body of stomach, intestinal carcinoma | 1 |
| C1629 | Cancer of the body of stomach, other or unspecified carcinoma | 1 |
| C1630 | Cancer of the pyloric orifice without histological confirmation | 1 |
| C1633 | Cancer of the pyloric orifice, diffuse carcinoma | 1 |
| C1634 | Cancer of the pyloric orifice, intestinal carcinoma | 1 |
| C168 | Gastric cancer extending to various regions | 1 |
| C169 | Unspecified gastric cancer | 1 |
| D130 | Benign neoplasm of esophagus | 1 |
| K575 | Diverticular disease of both small and large intestine without perforation and abscess | 1 |
| K552 | Angiodysplasia of colon | 1 |
| K621 | Rectal polyp | 1 |
| K500 | Crohn's disease of small intestine | 1 |
| K501 | Crohn's disease of large intestine | 1 |
| K512 | Ulcerative (chronic) proctitis | 1 |
| K513 | Ulcerative (chronic) rectosigmoiditis | 1 |
| K529 | Noninfective gastroenteritis and colitis, unspecified | 1 |
| C1821 | Cancer of the ascending colon, invasive carcinoma | 1 |
| D122 | Benign neoplasm of ascending colon | 1 |
| D123 | Benign neoplasm of transverse colon | 1 |
| D128 | Benign neoplasm of rectum | 1 |
| D129 | Benign neoplasm of anus or anal canal | 1 |
| K289 | Gastrojejunal ulcer, unspecified as acute or chronic, without hemorrhage or perforation | 1 |
| K631 | Perforation of intestine (nontraumatic) | 1 |
| K559 | Vascular disorder of intestine, unspecified | 1 |

GIB, gastrointestinal bleeding.

**Supplementary Table 4.** Summary of ICD-10 code counts for Lower GIB group

| **ICD10** | **Description** | **Count** |
| --- | --- | --- |
| Total |  | 1022 |
| K625 | Hemorrhage of anus and rectum | 266 |
| K921 | Melena | 175 |
| K573 | Diverticular disease of large intestine without perforation and abscess | 126 |
| K922 | Gastrointestinal hemorrhage, unspecified | 118 |
| D500 | Iron deficiency anaemia secondary to blood loss | 87 |
| D126 | Benign neoplasm of unspecified part of colon | 15 |
| C2091 | Cancer of rectum, invasive carcinoma | 14 |
| K519 | Ulcerative colitis, unspecified | 11 |
| D62 | Acute posthaemorrhagic anaemia | 11 |
| K920 | Hematemesis | 11 |
| K627 | Radiation proctitis | 10 |
| D128 | Benign neoplasm of rectum | 10 |
| K552 | Angiodysplasia of colon | 8 |
| C2090 | Cancer of rectum without histological confirmation | 8 |
| D374 | Neoplasm of uncertain or unknown behavior of colon | 7 |
| K284 | Chronic or unspecified gastrojejunal ulcer with hemorrhage | 7 |
| K579 | Diverticular disease of intestine, part unspecified, without perforation and abscess | 6 |
| D122 | Benign neoplasm of ascending colon | 6 |
| K280 | Acute gastrojejunal ulcer with hemorrhage | 6 |
| C1820 | Cancer of the ascending colon without histological confirmation | 5 |
| D125 | Benign neoplasm of sigmoid colon | 5 |
| K621 | Rectal polyp | 4 |
| K622 | Anal prolapse | 4 |
| K529 | Noninfective gastroenteritis and colitis, unspecified | 4 |
| C1871 | Cancer of sigmoid colon, invasive carcinoma | 4 |
| K558 | Other vascular disorders of intestine | 4 |
| K572 | Diverticular disease of large intestine with perforation and abscess | 3 |
| C1801 | Cancer of the cecum, invasive carcinoma | 3 |
| C1809 | Cancer of the cecum, other or unspecified histology | 3 |
| C1899 | Unspecified colon cancer, other or unspecified histology | 3 |
| D120 | Benign neoplasm of cecum | 3 |
| D124 | Benign neoplasm of descending colon | 3 |
| D129 | Benign neoplasm of anus or anal canal | 3 |
| D375 | Neoplasm of uncertain or unknown behavior of rectum | 3 |
| K286 | Chronic or unspecified gastrojejunal ulcer with both hemorrhage and perforation | 3 |
| K221 | Ulcer of esophagus | 2 |
| K254 | Chronic or unspecified gastric ulcer with hemorrhage | 2 |
| K210 | Gastro-oesophageal reflux disease with oesophagitis | 2 |
| K570 | Diverticular disease of small intestine with perforation and abscess | 2 |
| K629 | Disease of anus and rectum, unspecified | 2 |
| K508 | Other Crohn's disease | 2 |
| C1802 | Cancer of the cecum, carcinoma in adenoma | 2 |
| C1831 | Cancer of the right flexure of colon, invasive carcinoma | 2 |
| C1870 | Cancer of sigmoid colon without histological confirmation | 2 |
| C1879 | Cancer of sigmoid colon, other or unspecified histology | 2 |
| C2092 | Cancer of rectum, carcinoma in adenoma | 2 |
| C2099 | Cancer of rectum, other or unspecified histology | 2 |
| D127 | Benign neoplasm of rectosigmoid junction | 2 |
| D372 | Neoplasm of uncertain or unknown behavior of small intestine | 2 |
| I850 | Esophageal varices with bleeding | 1 |
| K293 | Chronic superficial gastritis | 1 |
| K295 | Unspecified chronic gastritis | 1 |
| K574 | Diverticular disease of both small and large intestine with perforation and abscess | 1 |
| K600 | Acute anal fissure | 1 |
| K620 | Anal polyp | 1 |
| K623 | Rectal prolapse | 1 |
| K626 | Ulcer of anus and rectum | 1 |
| K628 | Other specified diseases of anus and rectum | 1 |
| K500 | Crohn's disease of small intestine | 1 |
| K512 | Ulcerative (chronic) proctitis | 1 |
| K513 | Ulcerative (chronic) rectosigmoiditis | 1 |
| K515 | Mucosal proctocolitis | 1 |
| C172 | Cancer of the ileum | 1 |
| C1800 | Cancer of the cecum without histological confirmation | 1 |
| C1821 | Cancer of the ascending colon, invasive carcinoma | 1 |
| C1822 | Cancer of the ascending colon, carcinoma in adenoma | 1 |
| C1829 | Cancer of the ascending colon, other or unspecified histology | 1 |
| C1830 | Cancer of the right flexure of colon without histological confirmation | 1 |
| C1841 | Cancer of the transverse colon, invasive carcinoma | 1 |
| C1850 | Cancer of the left flexure of colon without histological confirmation | 1 |
| C1851 | Cancer of the left flexure of colon, invasive carcinoma | 1 |
| C1861 | Cancer of descending colon, invasive carcinoma | 1 |
| C1862 | Cancer of descending colon, carcinoma in adenoma | 1 |
| C1880 | Colon cancer extending to various regions without histological confirmation | 1 |
| C1890 | Unspecified colon cancer without histological confirmation | 1 |
| C1891 | Unspecified colon cancer, invasive carcinoma | 1 |
| C19 | Cancer of rectosigmoid junction | 1 |
| C210 | Unspecified anal cancer | 1 |
| D121 | Benign neoplasm of appendix | 1 |
| D123 | Benign neoplasm of transverse colon | 1 |
| K635 | Polyp of colon | 1 |
| K289 | Gastrojejunal ulcer, unspecified as acute or chronic, without hemorrhage or perforation | 1 |
| K631 | Perforation of intestine (nontraumatic) | 1 |
| K550 | Acute vascular disorder of intestine | 1 |

GIB, gastrointestinal bleeding.

**Supplementary Table 5.** Summary of ICD-10 code counts for Obscure GIB group

| **ICD10** | **Description** | **Count** |
| --- | --- | --- |
| Total |  | 3486 |
| K921 | Melena | 1032 |
| D500 | Iron deficiency anaemia secondary to blood loss | 778 |
| K625 | Hemorrhage of anus and rectum | 625 |
| K922 | Gastrointestinal hemorrhage, unspecified | 403 |
| K920 | Hematemesis | 401 |
| D62 | Acute posthaemorrhagic anaemia | 183 |
| K573 | Diverticular disease of large intestine without perforation and abscess | 22 |
| K558 | Other vascular disorders of intestine | 8 |
| K550 | Acute vascular disorder of intestine | 7 |
| K210 | Gastro-oesophageal reflux disease with oesophagitis | 6 |
| K295 | Unspecified chronic gastritis | 3 |
| K631 | Perforation of intestine (nontraumatic) | 2 |
| K559 | Vascular disorder of intestine, unspecified | 2 |
| K253 | Acute gastric ulcer without hemorrhage or perforation | 1 |
| K257 | Chronic gastric ulcer without hemorrhage or perforation | 1 |
| K263 | Acute duodenal ulcer without hemorrhage or perforation | 1 |
| K293 | Chronic superficial gastritis | 1 |
| K296 | Other gastritis | 1 |
| D131 | Benign neoplasm of stomach | 1 |
| K570 | Diverticular disease of small intestine with perforation and abscess | 1 |
| K579 | Diverticular disease of intestine, part unspecified, without perforation and abscess | 1 |
| K552 | Angiodysplasia of colon | 1 |
| K621 | Rectal polyp | 1 |
| K529 | Noninfective gastroenteritis and colitis, unspecified | 1 |
| D126 | Benign neoplasm of unspecified part of colon | 1 |
| D374 | Neoplasm of uncertain or unknown behavior of colon | 1 |
| K289 | Gastrojejunal ulcer, unspecified as acute or chronic, without hemorrhage or perforation | 1 |

GIB, gastrointestinal bleeding.

**Supplementary Table 6.** Summary of NCSP code counts for Upper GIB group. Among the group, 1422 patients (54.5%) underwent an endoscopic procedure during the GIB episode.

| **NCSP** | **Procedure** | **Count** |
| --- | --- | --- |
| Total |  | 1564 |
| UJD10 | Esophagoscopy, gastroscopy and duodenoscopy | 1346 |
| UJD02 | Gastroscopy | 73 |
| UJF32 | Coloscopy | 33 |
| JDA42 | Other endoscopic haemostatic procedure in stomach or pylorus | 27 |
| JDA32 | Endoscopic injection in stomach or pylorus | 22 |
| JCA42 | Other endoscopic procedure for hemostasis | 12 |
| JDA35 | Endoscopic contact coagulation in stomach or pylorus | 11 |
| JCA32 | Endoscopic injection in oesophagus | 8 |
| JDA52 | Other endoscopic procedure using diathermy or heat in stomach or pylorus | 8 |
| JDA22 | Endoscopic ligature of varices of stomach | 7 |
| JCA22 | Endoscopic ligature of oesophageal varices | 6 |
| UJF45 | Flexible sigmoidoscopy with biopsy | 4 |
| JFA32 | Other endoscopic haemostatic procedure in small intestine | 2 |
| JDA60 | Closure of perforated ulcer of stomach | 2 |
| JFA35 | Other endoscopic procedure using diathermy or heat in small intestine | 1 |
| UJF30 | Ileoscopy through colon | 1 |
| UJG02 | Endoscopy of rectum | 1 |

NCSP, Nordic Medico-Statistical Committee Classification of Surgical Procedures; GIB, gastrointestinal bleeding.

**Supplementary Table 7.** Summary of NCSP code counts for Lower GIB group. Among the group, 465 patients (69.5%) underwent an endoscopic procedure during the GIB episode.

| **NCSP** | **Procedure** | **Count** |
| --- | --- | --- |
| Total |  | 499 |
| UJF32 | Coloscopy | 326 |
| UJF45 | Flexible sigmoidoscopy with biopsy | 78 |
| UJF30 | Ileoscopy through colon | 28 |
| UJD10 | Esophagoscopy, gastroscopy and duodenoscopy | 23 |
| UJF92 | Capsular endoscopy | 17 |
| UJG02 | Endoscopy of rectum | 8 |
| JFA45 | Endoscopic contact coagulation in colon | 3 |
| JFA52 | Other endoscopic haemostatic procedure in colon | 3 |
| JFA55 | Other endoscopic procedure using diathermy or heat in colon | 3 |
| JFA32 | Other endoscopic haemostatic procedure in small intestine | 2 |
| JFA25 | Endoscopic contact coagulation in small intestine | 1 |
| JFA42 | Endoscopic injection in colon | 1 |
| UJF02 | Peroral enteroscopy | 1 |
| UJF12 | Enteroscopy through intestinal stoma | 1 |
| UJF34 | Left colonoscopy | 1 |
| JGA28 | Endoscopic contact coagulation in rectum | 1 |
| JGA32 | Proctotomy and local operations on rectum | 1 |
| UJD02 | Gastroscopy | 1 |

NCSP, Nordic Medico-Statistical Committee Classification of Surgical Procedures; GIB, gastrointestinal bleeding.

**Supplementary Table 8.** Summary of NCSP code counts for Obscure GIB group. Among the group, 61 patients (1.8%) underwent an endoscopic procedure during the GIB episode.

| **NCSP** | **Procedure** | **Count** |
| --- | --- | --- |
| Total |  | 121 |
| UJD10 | Esophagoscopy, gastroscopy and duodenoscopy | 59 |
| UJF32 | Coloscopy | 37 |
| UJF30 | Ileoscopy through colon | 9 |
| UJF45 | Flexible sigmoidoscopy with biopsy | 8 |
| UJD02 | Gastroscopy | 6 |
| UJF92 | Capsular endoscopy | 2 |

NCSP, Nordic Medico-Statistical Committee Classification of Surgical Procedures; GIB, gastrointestinal bleeding.

**Supplementary Table 9.** Incidence of oral anticoagulation initiation and competing risk of death within 90 days after atrial fibrillation diagnosis, stratified by gastrointestinal bleeding status, with subdistribution hazard ratios (Fine-Gray model). Patients with missing data were excluded from the regression analysis (N = 92,544).

| **Group** | **OAC initiations**  **n (%)** | **Deaths**  **n (%)** | **No events**  **n (%)** | **Unadjusted SHR (95% CI)** | **Adjusted SHR (95% CI)** |
| --- | --- | --- | --- | --- | --- |
| No prior GIB | 54,736 (62.7%) | 4,545 (5.2%) | 27,970 (32.1%) | Reference | Reference |
| GIB | 2,574 (48.6%) | 552 (10.4%) | 2,167 (40.9%) | 0.78 (0.76 - 0.81) | 0.84 (0.82 - 0.86) |

Abbreviations: OAC; oral anticoagulation, SHR, subdistribution hazard ratio; CI, confidence interval; GIB, gastrointestinal bleeding. SHRs were estimated using Fine–Gray subdistribution hazard models with all-cause death treated as a competing event. The adjusted analyses included the following covariates: age, sex, annual income, hypertension, hyperlipidemia, diabetes, coronary heart disease, congestive heart failure, prior ischemic stroke or transient ischemic attack, venous thromboembolism, dementia, abnormal liver function, psychiatric disease, alcohol abuse, use of antiplatelets or nonsteroidal anti-inflammatory drugs, selective serotonin reuptake inhibitors, proton pump inhibitors, active cancer, history of cancer, cancer site (upper vs. lower gastrointestinal tract), hemoglobin level, platelet count, and estimated glomerular filtration rate.

**Supplementary Table 10.** Factors associated with oral anticoagulation treatment after new-onset atrial fibrillation among patients without prior gastrointestinal bleeding. Patients with missing data were excluded from the modified Poisson regression analysis. All the displayed variables were forced into the model.

| **Variable** | **Study cohort**  **(n = 87,251)** | **Number of cases** | **Adjusted RR (95% CI)** |
| --- | --- | --- | --- |
| **Age, years** |  |  |  |
| Over 75 years | 48,145 | 30,535 | 1.39 (1.36 - 1.42) |
| 65-75 years | 26,414 | 18,011 | 1.39 (1.36 - 1.41) |
| Under 65 years | 12,692 | 6,190 | Reference |
| **Sex** |  |  |  |
| Male | 36,484 | 23,513 | 1.07 (1.06 - 1.08) |
| Female | 50,767 | 31,223 | Reference |
| **Annual income**^1^ |  |  |  |
| Quartile 4 (highest) | 21,342 | 13,214 | 1.04 (1.02 - 1.05) |
| Quartile 3 | 21,761 | 13,966 | 1.07 (1.05 - 1.08) |
| Quartile 2 | 20,941 | 13,689 | 1.08 (1.06 - 1.09) |
| Quartile 1 (lowest) | 23,207 | 13,867 | Reference |
| **Comorbidities** |  |  |  |
| Hypertension | 72,057 | 46,004 | 1.06 (1.04 - 1.07) |
| Hyperlipidemia | 49,751 | 33,139 | 1.13 (1.12 - 1.14) |
| Diabetes | 23,397 | 15,406 | 1.06 (1.05 - 1.07) |
| Coronary heart disease | 23,387 | 14,549 | 0.94 (0.93 - 0.96) |
| Congestive heart failure | 17,268 | 10,605 | 1.03 (1.02 - 1.05) |
| Ischemic stroke or TIA | 16,657 | 10,422 | 0.98 (0.96 - 0.99) |
| Venous thromboembolism | 6,909 | 4,343 | 1.02 (1.00 - 1.04) |
| Dementia | 5,490 | 2,443 | 0.71 (0.68 - 0.73) |
| Abnormal liver function | 430 | 173 | 0.76 (0.68 - 0.84) |
| Psychiatric disease | 13,416 | 7,603 | 0.99 (0.97 - 1.00) |
| Alcohol abuse | 3,179 | 1,497 | 0.80 (0.77 - 0.83) |
| **Medication** |  |  |  |
| Antiplatelets or NSAIDs | 27,264 | 17,417 | 1.03 (1.01 - 1.04) |
| SSRIs | 5,242 | 2,920 | 0.94 (0.92 - 0.96) |
| PPIs | 47,576 | 29,563 | 0.99 (0.98 - 1.00) |
| **Cancer status** |  |  |  |
| Current cancer | 4,453 | 1,825 | 0.66 (0.64 - 0.69) |
| Previous cancer | 17,352 | 10,905 | 0.97 (0.95 - 0.98) |
| Lower GI tract cancer | 1,484 | 818 | 0.98 (0.94 - 1.03) |
| Upper GI tract cancer | 265 | 96 | 0.73 (0.63 - 0.85) |
| **Hemoglobin level**^2^ |  |  |  |
| Anemia | 22,648 | 12,286 | 0.85 (0.84 - 0.86) |
| Normal hemoglobin | 64,603 | 42,450 | Reference |
| **Platelet count** (mcL)^3^ |  |  |  |
| Under 100 | 1,363 | 500 | 0.67 (0.62 - 0.72) |
| Over 100 | 85,888 | 54,236 | Reference |
| **eGFR** (ml/min/1.73m^2^)^4^ |  |  |  |
| <15 | 834 | 296 | 0.61 (0.55 - 0.67) |
| 15-29 | 2,315 | 1,073 | 0.76 (0.73 - 0.79) |
| 30-59 | 21,864 | 13,820 | 0.98 (0.97 - 0.99) |
| >60 | 62,238 | 39,547 | Reference |

Abbreviations: RR, relative risk; CI, confidence interval; TIA, transient ischemic attack; NSAID, nonsteroidal anti-inflammatory drug; SSRI, selective serotonin reuptake inhibitor; PPI, proton-pump inhibitor; GI, gastrointestinal; eGFR, estimated glomerular filtration rate.

^1^Data was missing for 0.01% (n = 11)

^2^Data was missing for 18.2% (n = 21,435)

^3^Data was missing for 18.2% (n = 21,462)

^4^Data was missing for 19.8% (n = 23,314)

**Supplementary Table 11.** Factors associated with oral anticoagulation initiation among patients with gastrointestinal bleeding (GIB), excluding ICD-10 codes D50.0 and D62 from the GIB definition. Patients with missing data were excluded from the modified Poisson regression analysis. All the displayed variables were forced into the model.

| **Variable** | **Valid n**  **()** | **Number of cases** | **Adjusted RR**  **(95% CI)** |
| --- | --- | --- | --- |
| **Age, years** |  |  |  |
| Over 75 years | 3,461 | 1,712 | 1.40 (1.23 - 1.59) |
| 65-75 years | 1,379 | 694 | 1.30 (1.14 - 1.49) |
| Under 65 years | 453 | 168 | Reference |
| **Sex** |  |  |  |
| Male | 2,583 | 1,254 | 1.07 (1.01 - 1.13) |
| Female | 2,710 | 1,320 | Reference |
| **Annual income**^1^ |  |  |  |
| Quartile 4 (highest) | 1,296 | 659 | 1.12 (1.04 - 1.22) |
| Quartile 3 | 1,254 | 642 | 1.10 (1.02 - 1.19) |
| Quartile 2 | 1,226 | 601 | 1.10 (1.02 - 1.19) |
| Quartile 1 (lowest) | 1,517 | 672 | Reference |
| **Comorbidities** |  |  |  |
| Hypertension | 4,597 | 2,273 | 1.06 (0.97 - 1.16) |
| Hyperlipidemia | 3,188 | 1,690 | 1.20 (1.12 - 1.28) |
| Diabetes | 1,707 | 847 | 1.05 (0.99 - 1.12) |
| Coronary heart disease | 1,995 | 979 | 0.98 (0.92 - 1.04) |
| Congestive heart failure | 1,603 | 719 | 0.96 (0.90 - 1.03) |
| Ischemic stroke or TIA | 1,246 | 591 | 0.91 (0.85 - 0.97) |
| Venous thromboembolism | 614 | 274 | 0.97 (0.89 - 1.07) |
| Dementia | 479 | 156 | 0.66 (0.58 - 0.76) |
| Abnormal liver function | 168 | 34 | 0.50 (0.36 - 0.71) |
| Psychiatric disease | 1,189 | 501 | 1.05 (0.96 - 1.13) |
| Alcohol abuse | 496 | 160 | 0.69 (0.59 - 0.80) |
| **Medication** |  |  |  |
| Antiplatelet or NSAID | 1,615 | 833 | 1.07 (1.01 - 1.14) |
| SSRI | 388 | 167 | 0.99 (0.88 - 1.11) |
| PPI | 4,392 | 2,185 | 1.07 (0.98 - 1.17) |
| **Cancer status** |  |  |  |
| Current cancer | 408 | 137 | 0.73 (0.63 - 0.85) |
| Previous cancer | 1,360 | 667 | 0.96 (0.90 - 1.03) |
| Lower GI tract cancer | 268 | 128 | 1.03 (0.90 - 1.19) |
| Upper GI tract cancer | 79 | 26 | 0.73 (0.52 - 1.01) |
| **Hemoglobin level**^2^ |  |  |  |
| Anemia | 2,551 | 978 | 0.72 (0.68 - 0.77) |
| Normal hemoglobin | 2,742 | 1,596 | Reference |
| **Platelet count** (mcL)^3^ |  |  |  |
| Under 100 | 178 | 30 | 0.46 (0.32 - 0.67) |
| Over 100 | 5,115 | 2,544 | Reference |
| **eGFR** (ml/min/1.73m^2^)^4^ |  |  |  |
| <15 | 120 | 26 | 0.48 (0.32 - 0.71) |
| 15-29 | 306 | 103 | 0.69 (0.58 - 0.82) |
| 30-59 | 1,655 | 809 | 0.96 (0.90 - 1.02) |
| >60 | 3,212 | 1,636 | Reference |
| **GIB subgroup** |  |  |  |
| Lower | 519 | 283 | 0.99 (0.91 - 1.08) |
| Obscure | 2,695 | 1,239 | 0.93 (0.88 - 0.99) |
| Upper | 2,079 | 1,052 | Reference |
| **Time between GIB and AF diagnosis** |  |  |  |
| GIB temporally with AF (± 30 days) | 1,037 | 348 | 0.72 (0.65 - 0.80) |
| Prior GIB (>30 days) | 4,256 | 2,226 | Reference |

Abbreviations: GIB, gastrointestinal bleeding; RR, relative risk; CI, confidence interval; TIA, transient ischemic attack; NSAID, nonsteroidal anti-inflammatory drug; SSRI, selective serotonin reuptake inhibitor; PPI, proton-pump inhibitor; eGFR, estimated glomerular filtration rate.

^1^Data was missing for 0.01% (n = 1)

^2^Data was missing for 18.8% (n = 1,079)

^3^Data was missing for 18.8% (n = 1,081

^4^Data was missing for 19.9% (n = 1,141)

**Supplementary Table 12.** Factors associated with oral anticoagulation initiation among patients with gastrointestinal bleeding (GIB), excluding those who experienced GIB after the diagnosis of new-onset atrial fibrillation. Patients with missing data were excluded from the modified Poisson regression analysis. All displayed variables were forced into the model.

| **Variable** | **Valid n** | **Number of cases** | **Adjusted RR**  **(95% CI)** |
| --- | --- | --- | --- |
| **Age, years** |  |  |  |
| Over 75 years | 3,202 | 1,584 | 1.37 (1.20 - 1.55) |
| 65-75 years | 1,296 | 652 | 1.29 (1.13 - 1.46) |
| Under 65 years | 428 | 161 | Reference |
| **Sex** |  |  |  |
| Male | 2,426 | 1,183 | 1.09 (1.03 - 1.15) |
| Female | 2,500 | 1,214 | Reference |
| **Annual income**^1^ |  |  |  |
| Quartile 4 (highest) | 1,216 | 621 | 1.12 (1.04 - 1.21) |
| Quartile 3 | 1,175 | 600 | 1.10 (1.02 - 1.19) |
| Quartile 2 | 1,140 | 557 | 1.09 (1.01 - 1.18) |
| Quartile 1 (lowest) | 1,395 | 619 | Reference |
| **Comorbidities** |  |  |  |
| Hypertension | 4,297 | 2,125 | 1.07 (0.97 - 1.17) |
| Hyperlipidemia | 2,984 | 1,582 | 1.19 (1.11 - 1.26) |
| Diabetes | 1,610 | 801 | 1.08 (1.01 - 1.14) |
| Coronary heart disease | 1,865 | 913 | 0.96 (0.90 - 1.02) |
| Congestive heart failure | 1,491 | 667 | 0.97 (0.91 - 1.04) |
| Ischemic stroke or TIA | 1,175 | 560 | 0.93 (0.87 - 1.00) |
| Venous thromboembolism | 586 | 260 | 0.94 (0.86 - 1.03) |
| Dementia | 446 | 140 | 0.63 (0.55 - 0.72) |
| Abnormal liver function | 165 | 34 | 0.57 (0.43 - 0.77) |
| Psychiatric disease | 1,121 | 469 | 1.02 (0.94 - 1.11) |
| Alcohol abuse | 472 | 154 | 0.71 (0.62 - 0.82) |
| **Medication** |  |  |  |
| Antiplatelet or NSAID | 1,506 | 784 | 1.09 (1.03 - 1.16) |
| SSRI | 357 | 151 | 0.94 (0.84 - 1.06) |
| PPI | 4,195 | 2,076 | 1.02 (0.94 - 1.11) |
| **Cancer status** |  |  |  |
| Current cancer | 393 | 129 | 0.71 (0.61 - 0.82) |
| Previous cancer | 1,264 | 627 | 0.98 (0.92 - 1.04) |
| Lower GI tract cancer | 264 | 125 | 1.04 (0.91 - 1.19) |
| Upper GI tract cancer | 78 | 26 | 0.76 (0.56 - 1.03) |
| **Hemoglobin level**^2^ |  |  |  |
| Anemia | 2,366 | 896 | 0.74 (0.70 - 0.79) |
| Normal hemoglobin | 2,560 | 1,501 | Reference |
| **Platelet count** (mcL)^3^ |  |  |  |
| Under 100 | 169 | 27 | 0.47 (0.34 - 0.67) |
| Over 100 | 4,757 | 2,370 | Reference |
| **eGFR** (ml/min/1.73m^2^)^4^ |  |  |  |
| <15 | 110 | 23 | 0.49 (0.35 - 0.70) |
| 15-29 | 283 | 93 | 0.70 (0.60 - 0.83) |
| 30-59 | 1,548 | 755 | 0.97 (0.91 - 1.03) |
| >60 | 2,985 | 1,526 | Reference |
| **GIB subgroup** |  |  |  |
| Lower | 505 | 277 | 0.99 (0.90 - 1.08) |
| Obscure | 2,448 | 1,109 | 0.90 (0.85 - 0.96) |
| Upper | 1,973 | 1,011 | Reference |
| **Time between GIB and AF diagnosis** |  |  |  |
| GIB temporally with AF (<30 days) | 617 | 147 | 0.54 (0.46 - 0.62) |
| Prior GIB (>30 days) | 4,309 | 2,250 | Reference |

Abbreviations: GIB, gastrointestinal bleeding; RR, relative risk; CI, confidence interval; TIA, transient ischemic attack; NSAID, nonsteroidal anti-inflammatory drug; SSRI, selective serotonin reuptake inhibitor; PPI, proton-pump inhibitor; eGFR, estimated glomerular filtration rate.

^1^Data was missing for 0.01% (n = 1)

^2^Data was missing for 18.7% (n = 1,151)

^3^Data was missing for 18.7% (n = 1,155)

^4^Data was missing for 19.7% (n = 1,215)

**Supplementary Table 13.** Factors associated with oral anticoagulation treatment after new-onset atrial fibrillation in patients with gastrointestinal bleeding (GIB) without the attributes of GIB (subgroup, temporal proximity). Patients with missing data were excluded from the modified Poisson regression analysis. All the displayed variables were forced into the model.

| **Variable** | **GIB**  **(n = 5,293)** | **Number of cases** | **Adjusted RR (95% CI)** |
| --- | --- | --- | --- |
| **Age, years** |  |  |  |
| Over 75 years | 3,461 | 1,712 | 1.39 (1.36 - 1.42) |
| 65-75 years | 1,379 | 694 | 1.39 (1.36 - 1.41) |
| Under 65 years | 453 | 168 | Reference |
| **Sex** |  |  |  |
| Male | 2,583 | 1,254 | 1.07 (1.06 - 1.08) |
| Female | 2,710 | 1,320 | Reference |
| **Annual income**^1^ |  |  |  |
| Quartile 4 (highest) | 1,296 | 659 | 1.04 (1.02 - 1.05) |
| Quartile 3 | 1,254 | 642 | 1.07 (1.05 - 1.08) |
| Quartile 2 | 1,226 | 601 | 1.08 (1.06 - 1.09) |
| Quartile 1 (lowest) | 1,517 | 672 | Reference |
| **Comorbidities** |  |  |  |
| Hypertension | 4,597 | 2,273 | 1.06 (1.04 - 1.07) |
| Hyperlipidemia | 3,188 | 1,690 | 1.13 (1.12 - 1.14) |
| Diabetes | 1,707 | 847 | 1.06 (1.05 - 1.07) |
| Coronary heart disease | 1,995 | 979 | 0.94 (0.93 - 0.96) |
| Congestive heart failure | 1,603 | 719 | 1.03 (1.02 - 1.05) |
| Ischemic stroke or TIA | 1,246 | 591 | 0.98 (0.96 - 0.99) |
| Venous thromboembolism | 614 | 274 | 1.02 (1.00 - 1.04) |
| Dementia | 479 | 156 | 0.71 (0.68 - 0.73) |
| Abnormal liver function | 168 | 34 | 0.76 (0.68 - 0.84) |
| Psychiatric disease | 1,189 | 501 | 0.99 (0.97 - 1.00) |
| Alcohol abuse | 496 | 160 | 0.80 (0.77 - 0.83) |
| **Medication** |  |  |  |
| Antiplatelets or NSAIDs | 1,615 | 833 | 1.03 (1.01 - 1.04) |
| SSRIs | 388 | 167 | 0.94 (0.92 - 0.96) |
| PPIs | 4,392 | 2,185 | 0.99 (0.98 - 1.00) |
| **Cancer status** |  |  |  |
| Current cancer | 408 | 137 | 0.66 (0.64 - 0.69) |
| Previous cancer | 1,360 | 667 | 0.97 (0.95 - 0.98) |
| Lower GI tract cancer | 268 | 128 | 0.98 (0.94 - 1.03) |
| Upper GI tract cancer | 79 | 26 | 0.73 (0.63 - 0.85) |
| **Hemoglobin level**^2^ |  |  |  |
| Anemia | 2,551 | 978 | 0.85 (0.84 - 0.86) |
| Normal hemoglobin | 2,742 | 1,596 | Reference |
| **Platelet count** (mcL)^3^ |  |  |  |
| Under 100 | 178 | 30 | 0.67 (0.62 - 0.72) |
| Over 100 | 5,115 | 2,544 | Reference |
| **eGFR** (ml/min/1.73m^2^)^4^ |  |  |  |
| <15 | 120 | 26 | 0.61 (0.55 - 0.67) |
| 15-29 | 306 | 103 | 0.76 (0.73 - 0.79) |
| 30-59 | 1,655 | 809 | 0.98 (0.97 - 0.99) |
| >60 | 3,212 | 1,636 | Reference |

Abbreviations: GIB, gastrointestinal bleeding; RR, relative risk; CI, confidence interval; TIA, transient ischemic attack; NSAID, nonsteroidal anti-inflammatory drug; SSRI, selective serotonin reuptake inhibitor; PPI, proton-pump inhibitor; eGFR, estimated glomerular filtration rate.

^1^Data was missing for 0.01% (n = 1)

^2^Data was missing for 18.7% (n = 1,240)

^3^Data was missing for 18.7% (n = 1,244)

^4^Data was missing for 19.7% (n = 1,309)

**Supplementary Table 14.** Summary of the time gap between baseline laboratory testing date and cohort entry date

| **Laboratory test** | **365 days** | **180 days** | **30 days** | **7 days** |
| --- | --- | --- | --- | --- |
| Hemoglobin | 99.0% | 94.8% | 84.9% | 77.6% |
| Platelet count | 99.0% | 94.8% | 84.9% | 77.5% |
| eGFR | 96.9% | 92.1% | 81.1% | 73.4% |

Abbreviations: eGFR, estimated glomerular filtration rate calculated from serum creatinine level using the CKD-EPI equation.

The table displays the portion of the study cohort (96,135 patients [81.5%]) that had at least one available result in the laboratory tests being examined (either hemoglobin, platelet count, or creatinine) before cohort entry. The proportion of patients with available results for the respective laboratory tests at this time point before cohort entry is presented.

**Supplementary Table 15.** Comparison of baseline characteristics between patients included in the regression analysis and those excluded due to missing data

|  |  |  |
| --- | --- | --- |
| **Variable** | **Cases with complete data** | **Cases with missing data** |
|  | (n = 92,544) | (n = 25,453) |
| **Demographics and socioeconomic status** |  |  |
| Age, years | 75.7 (10.9) | 74.6 (11.2) |
| Male sex | 39,067 (42.2) | 10,854 (42.6) |
| Annual taxable income, 1000 € | 13.0 (4.0 - 29.0) | 13.0 (4.0 - 29.0) |
| **Risk scores** |  |  |
| CHA_2_DS_2_-VASc score | 4.0 (1.6) | 3.8 (1.5) |
| Modified HAS-BLED score | 2.4 (1.0) | 2.3 (1.0) |
| **Comorbidities** |  |  |
| Prior bleeding | 11,932 (12.9) | 2,832 (11.1) |
| Hypertension | 76,654 (82.8) | 20,723 (81.4) |
| Hyperlipidemia | 52,939 (57.2) | 13,552 (53.2) |
| Diabetes | 25,104 (27.1) | 6,234 (24.5) |
| Coronary heart disease | 25,382 (27.4) | 5,990 (23.5) |
| Congestive heart failure | 18,871 (20.4) | 4,255 (16.7) |
| Ischemic stroke or TIA | 17,903 (19.3) | 4,099 (16.1) |
| Venous thromboembolism | 7,523 (8.1) | 1,757 (6.9) |
| Dementia | 5,969 (6.4) | 1,064 (4.2) |
| Abnormal kidney function | 4,880 (5.3) | 1,101 (4.3) |
| Abnormal liver function | 598 (0.6) | 110 (0.4) |
| Psychiatric disease | 14,605 (15.8) | 3,108 (12.2) |
| Alcohol abuse | 3,675 (4.0) | 828 (3.3) |
| **Medication** |  |  |
| Antiplatelet or NSAID | 28,879 (31.2) | 7,606 (29.9) |
| SSRI | 5,630 (6.1) | 1,329 (5.2) |
| PPI | 51,968 (56.2) | 12,999 (51.1) |
| **Cancer status** |  |  |
| Current cancer | 4,861 (5.3) | 873 (3.4) |
| Previous cancer | 18,712 (20.2) | 4,669 (18.3) |
| Lower GI tract cancer | 1,752 (1.9) | 398 (1.6) |
| Upper GI tract cancer | 344 (0.4) | 65 (0.3) |
| **Laboratory results** |  |  |
| Hemoglobin (g/l) | 133 (18) | 138 (16) |
| Platelet count (mcL) | 238 (84) | 230 (72) |
| eGFR (ml/min/1.73m^2^) | 70 (21) | 73 (19) |

*Note*: Quantitative variables are presented as mean (standard deviation), except for annual income, which is non-normally distributed and is presented as median (interquartile range). Categorical variables are presented as count (percentage).

Abbreviations: GIB: gastrointestinal bleeding; CHA_2_DS_2_-VASc, congestive heart failure, hypertension, age ≥75 years, diabetes, history of stroke or TIA, vascular disease, age 65–74 years, sex category (female); Modified HAS-BLED score, hypertension, abnormal renal or liver function, prior stroke, bleeding history, age >65 years, alcohol abuse, concomitant antiplatelet/NSAIDs (no labile INR, max score 8); TIA, transient ischemic attack; NSAID, nonsteroidal anti-inflammatory drug; SSRI, selective serotonin reuptake inhibitor; PPI, proton-pump inhibitor; eGFR, estimated glomerular filtration rate.

**Supplementary references**

1. Camaschella C. Iron-deficiency anemia. N Engl J Med. 2015 May 7;372(19):1832–43.

2. Kumar A, Sharma E, Marley A, Samaan MA, Brookes MJ. Iron deficiency anaemia: pathophysiology, assessment, practical management. BMJ Open Gastroenterol. 2022 Jan;9(1).
